# Structural and functional studies of PCNA from African swine fever virus

**DOI:** 10.1128/jvi.00748-23

**Published:** 2023-08-03

**Authors:** Zhiwei Shao, Jie Yang, Yanqing Gao, Yixi Zhang, Xin Zhao, Qiyuan Shao, Weizhen Zhang, Chulei Cao, Hehua Liu, Jianhua Gan

**Affiliations:** 1 Shanghai Public Health Clinical Center, State Key Laboratory of Genetic Engineering, Collaborative Innovation Center of Genetics and Development, School of Life Sciences, Fudan University, Shanghai, China; University of Toronto, Toronto, Ontario, Canada

**Keywords:** African swine fever virus, ASFV, proliferating cell nuclear antigen, PCNA, DNA ligase, crystal structure, DNA replication, DNA repair, DNA binding, DNA ligation, drug target

## Abstract

Proliferating cell nuclear antigen (PCNA) belongs to the DNA sliding clamp family. Via interacting with various partner proteins, PCNA plays critical roles in DNA replication, DNA repair, chromatin assembly, epigenetic inheritance, chromatin remodeling, and many other fundamental biological processes. Although PCNA and PCNA-interacting partner networks are conserved across species, PCNA of a given species is rarely functional in heterologous systems, emphasizing the importance of more representative PCNA studies. Here, we report two crystal structures of PCNA from African swine fever virus (ASFV), which is the only member of the *Asfarviridae* family. Compared to the eukaryotic and archaeal PCNAs and the sliding clamp structural homologs from other viruses, *Asfv*PCNA possesses unique sequences and/or conformations at several regions, such as the J-loop, interdomain-connecting loop (IDCL), P-loop, and C-tail, which are involved in partner recognition or modification of sliding clamps. In addition to double-stranded DNA binding, we also demonstrate that *Asfv*PCNA can modestly enhance the ligation activity of the *Asfv*LIG protein. The unique structural features of *Asfv*PCNA can serve as a potential target for the development of ASFV-specific inhibitors and help combat the deadly virus.

**IMPORTANCE:**

Two high-resolution crystal structures of African swine fever virus proliferating cell nuclear antigen (*Asfv*PCNA) are presented here. Structural comparison revealed that *Asfv*PCNA is unique at several regions, such as the J-loop, the interdomain-connecting loop linker, and the P-loop, which may play important roles in ASFV-specific partner selection of *Asfv*PCNA. Unlike eukaryotic and archaeal PCNAs, *Asfv*PCNA possesses high double-stranded DNA-binding affinity. Besides DNA binding, *Asfv*PCNA can also modestly enhance the ligation activity of the *Asfv*LIG protein, which is essential for the replication and repair of ASFV genome. The unique structural features make *Asfv*PCNA a potential target for drug development, which will help combat the deadly virus.

## INTRODUCTION

As one of the most fundamental biological processes, DNA replication transfers genetic information and ensures the integrity of the genome. Different from the continuous synthesis of the leading strand, the DNA lagging strand is replicated in a discontinuous fashion, forming multiple short fragments (known as Okazaki fragment). Synthesis of each fragment requires a primer that is produced by the PrimPol family proteins. The bulk synthesis of the leading and lagging strands is catalyzed by replicative polymerases, such as Pol ε and Pol δ in eukaryotes (1). Although sliding clamp family proteins have no enzymatic activities, they play important roles in DNA replication (2, 3). Sliding clamp family proteins are highly conserved in all three domains of life (4). The eukaryotic and archaeal sliding clamp family proteins are also known as proliferating cell nuclear antigens (PCNAs); each PCNA molecule has two similar domains (Domain I and Domain II), which are connected by one interdomain-connecting loop (IDCL). Different from PCNAs, β clamps (the bacteria sliding clamps) contain three similar domains. Both PCNA and β clamp typically form closed ring-shaped structures; their loading onto DNA is facilitated by other replication factors, such as replication factor C (RFC) in an ATP-dependent manner (5). Association with PCNA can enhance the activity of Pol δ by over 30-folds (6).

When the replication reaches the end from a previous Okazaki fragment, PCNA recruits the Fen-1 endonuclease that specifically recognizes and removes the flap DNA structure (7). The resulting nick is sealed by DNA ligase 1 (*Hs*LIG1) in human (8) or ligase Cdc9 in *Saccharomyces cerevisiae* (9, 10). In addition to *Hs*LIG1, human expresses three additional ligases, *Hs*LIG2–*HS*LIG4. Recruited by PCNA, these ligases catalyze the final nick sealing step in various DNA repair pathways (2), including mismatch repair (MMR), base excision repair (BER), and nucleotide excision repair (NER). Interestingly, PCNA can also interact with many other proteins that function in the early steps of DNA repair. For example, PCNA can recruit EXO1 that conducts strand excision in the MMR pathway. In the BER pathway, PCNA interacts with both uracil-DNA glycosylase 2 and AP endonuclease (11, 12). Via interacting with the endonuclease XPG (which cuts the strand 3′ to the damage), PCNA can significantly enhance the efficiency of the NER pathway *in vivo* (13).

In addition to DNA replication and DNA repair, PCNA also functions in many other fundamental biological processes, such as chromatin assembly (3, 14), epigenetic inheritance (15), chromatin remodeling (16, 17), cell cycle control (18), and apoptosis (19), via interacting with different partner proteins. Although it is already stunning, the number of PCNA-interacting proteins is still growing. As a result, no other known protein could rival PCNA in its ability to interact with so many different partners. PCNAs have been extensively studied in many species, such as human, rat, fly, yeast, *Arabidopsis,* and many archaea (20, 21). These studies showed that PCNA and PCNA-interacting partner network are conserved across species, but PCNA of a given species is rarely functional in heterologous systems (22). The structures of many PCNAs have been reported, including PCNAs from *Sulfolobus solfataricus (Ss*), human (*Hs*), *S. cerevisiae* (*Sc*), *Candida albicans* (*Ca*), *Neurospora crassa* (*Nc*), and several other fungi (23 - 25). These structures showed that PCNAs normally function as trimers, and the IDCL linkers play critical roles in species-specific polymerase (26, 27), ligase (28), or other partner recognition by PCNAs. Although β clamps differ from PCNAs in the domain numbers, the ring-shaped structures formed by β clamps and PCNAs are similar (29, 30). Like PCNAs, β clamps mainly use their IDCL linkers in partner interactions.

Due to their important biological functions, sliding clamp family proteins have been considered potential drug targets (31). In fact, various sliding clamp targeting compounds have been identified, such as the griselimycin class of antibiotics. By blocking the interactions between the sliding clamp and the catalytic subunit of the polymerase, griselimycins are highly active against *Mycobacterium tuberculosis* both *in vitro* and *in vivo* (32). To better understand the biological functions and facilitate species-specific drug development, more representative sliding clamp protein structures are needed. The structures of sliding clamp structural homologs from several human herpesviruses, including UL42 from the alphaherpesvirus herpes simplex virus type 1 (HSV-1), UL44 from the betaherpesvirus human cytomegalovirus (HCMV), BMRF1 from Epstein-Barr virus, and PF-8 from Kaposi’s sarcoma-associated herpesvirus (KSHV), have been reported (33 - 35), whereas the sliding clamp structures from many other pathogenic viruses are still unavailable, such as African swine fever virus (ASFV). ASFV is highly contagious and can cause lethal disease in pigs (36). ASFV was first discovered in the 1920s and remained restricted to Africa till the mid-1950s (37). Since then, ASFV has spread to many countries in Europe, South America, and Asia. In 2018, ASFV that was reported in China, the largest pork producer in the world, caused huge economic losses and an immediate pork shortage (38). Although it has been extensively studied for over 100 years, safe and effective vaccines against ASFV are still very limited (39). Instead, ASFV has become a global threat to the agricultural industry in recent years.

ASFV is a large double-stranded DNA (dsDNA) virus and is one of the most complex viruses known to date. The size of the ASFV genome varies between 170 and 190 kb, encoding more than 160 proteins that are involved in entry into host cells, suppression of host immune response (40), DNA replication (41), DNA damage response (42), DNA repair (43), and gene expression and virion assembly of ASFV (44). During replication, small viral DNA fragments are synthesized in the nucleus of the infected cells at early times, while large fragments are synthesized in the cytoplasm at later times. Both small and large DNA fragments are precursors of mature head-to-head cross-linked viral DNAs. Through site-specific nicking, rearrangement, and ligation, the cross-linked DNAs are resolved and form the genomic DNA of ASFV (45, 46). ASFV belongs to the Asfivirus genus and is the only member of the *Asfarviridae* family. Although the reported ASFV protein structures are still very limited, previous studies showed that the structures of ASFV proteins could be very different from their homologous proteins from other species (47 - 49). Here, we report the structural and biochemical studies of the *Asfv*PCNA protein. The crystal structures were determined at atomic resolution and confirmed that *Asfv*PCNA can assemble into a homotrimeric ring-shaped structure. Different from homologous protein structures, *Asfv*PCNA has a very unique amino acid sequence and conformation at the IDCL, J-loop, and P-loop regions, which are involved in partner recognition by PCNAs. Although the detailed molecular basis remains to be investigated, *Asfv*PCNA has a high dsDNA-binding affinity and can enhance the catalytic activity of the *Asfv*LIG protein, which plays critical roles in both DNA replication and DNA repair pathways of ASFV. Our study not only advances our understanding on the function of *Asfv*PCNA but also provides a potential target for anti-ASFV drug development.

## RESULTS

### Crystal structure of *Asfv*PCNA

*Asfv*PCNA is a hypothetic sliding clamp family protein; it is coded by the E301R gene that is highly conserved in ASFV strains. The high conservation suggests that *Asfv*PCNA may play certain functional role in ASFV; however, *Asfv*PCNA shares no clear sequence similarity with other sliding clamp family members, and the detailed function of *Asfv*PCNA has not been experimentally verified. To investigate the potential function of *Asfv*PCNA, we constructed and expressed one His-Sumo-tagged *Asfv*PCNA protein. After the His-Trap column purification, the tag was removed and the target protein was further purified by size-exclusion S200 16/600 column (Fig. S1A). Two protein peaks were observed: the peak 1 was eluted at the void volume of the column and the peak 2 was eluted at a volume of 78.2 mL, corresponding to the molecular weight of an *Asfv*PCNA homotrimer. The ratio between OD280 and OD260 is 1.75, suggested that the purified *Asfv*PCNA protein is free of nucleic acids. The purity of *Asfv*PCNA was confirmed by the SDS-PAGE gel analysis (Fig. S1B).

**Fig 1:**
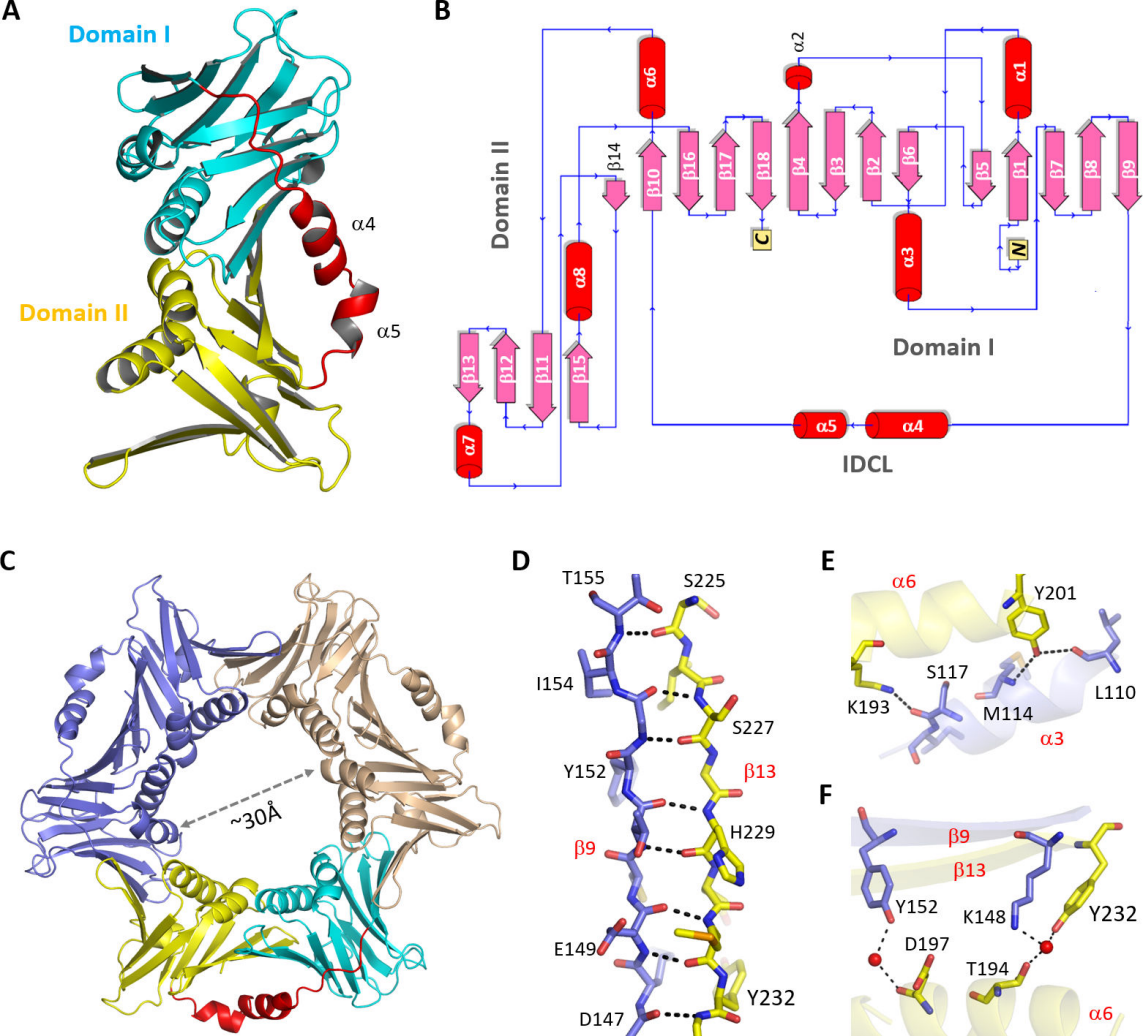
Crystal structure of *Asfv*PCNA. (**A**) Overall folding of *Asfv*PCNA observed in Form I structure. Domain I, Domain II, and IDCL linker are colored in cyan, yellow, and red, respectively. (**B**) Topology of *Asfv*PCNA. (**C**) Cartoon representation of the trimeric *Asfv*PCNA protein. One protomer is colored as in panel A, whereas the other two protomers are colored in wheat and light blue, respectively. (**D–F**) The detailed interactions involved in *Asfv*PCNA trimerization. The C-atoms of the neighboring protomers are colored in yellow and light blue, respectively.

Using the purified *Asfv*PCNA protein, we performed extensive crystallization trials and solved the structures in two different forms, Form I and Form II (Table 1). Form I structure was refined at higher resolution (2.2 Å); therefore, it was used for detailed structural analysis. Form I crystal belongs to P6_3_ space group, per asymmetric unit contains one *Asfv*PCNA molecule (Fig. 1A). Out of the total 301 residues of *Asfv*PCNA, residues 4–15 at the N-terminus and residues 298–301 at the very C-terminus are disordered. The remaining residues form 8 α-helices and 18 β-strands (Fig. S2), assembled into two domains, Domain I and Domain II (Fig. 1A and B). Domain I (residues 1–155) is composed of α1–3 and β1–9, and Domain II (residues 181–301) consisted of α6–8 and β10–18. The overall folding of the two domains is similar. All the β-strands are antiparallel (Fig. 1A) and reside in the middle of the structure; the six α-helices of Domain I and Domain II locate on the same side of the β-strands.

**TABLE 1:** Data collection and refinement statistics

Structure	Form I	Form II
PDB ID	7YPE	7YPF
Data collection^*a*^		
Space group	P6_3_	P1
Cell parameter:		
*α*, *b*, *c* (Å)	118.660, 118.660, 54.510	54.659, 118.873, 118.843
α, β, γ (°)	90.000, 90.000, 120.000	60.029, 89.978, 89.963
Wavelength (Å)	0.9793	0.9793
Resolution (Å)	29.66–2.20	30.0–2.50
High-resolution shell (Å)	2.27–2.20	2.59–2.50
Completeness (%)	100.0 (100.0)	97.5 (93.3)
Redundancy	13.6 (12.4)	3.3 (2.8)
*R*_merge_ (%)	11.9 (132.7)	5.2 (40.6)
*I*/σ (I)	18.9 (2.6)	19.8 (2.3)
Refinement		
Resolution (Å)	29.66–2.20	29.73–2.50
No. of reflections	22,445	84,025
*R*_work_ (%)/*R*_free_ (%)	20.9/22.7	20.8/23.3
No. of atoms		
Protein	2,338	13,830
Water	59	110
Root mean square deviations		
Bond length (Å)	0.002	0.004
Bond angle (°)	0.557	1.019
Ramachandran plot (%)		
Most favorable	96.5	96.6
Additional allowed	3.5	3.4
Outlier	0.00	0.0

^*a*^ Values in parentheses are for the high-resolution shell.

Similar to all reported PCNA structures, Domain I and Domain II of *Asfv*PCNA are connected by an IDCL linker (Fig. 1A and B). *Asfv*PCNA IDCL is composed of 25 residues, residues 156–180. Although the side chains of some *Asfv*PCNA IDCL residues are flexible, the main chains of the IDCL linker are well ordered in Form I structure, supported by the clear 2F_o_−F_c_ electron density maps (Fig. S3A). In addition to the loop structures at its two ends, *Asfv*PCNA IDCL also contains two short α-helices, α4 and α5, which are formed by residues 164–171 and 174–177, respectively.

Unlike the Form I structure, the Form II *Asfv*PCNA structure belongs to P1 space group (Table 1); per asymmetric unit contains six *Asfv*PCNA molecules, which assemble into two homotrimeric ring structures (Fig. S4). Via symmetric operation, Form I *Asfv*PCNA structure can also form homotrimer (Fig. 1C). The overall conformations of Form I and Form II *Asfv*PCNA trimers are virtually identical, supported by the very low root mean square deviation (RMSD, 0.2 Å) value over 840 pairs of Cα atoms. The three *Asfv*PCNA protomers arrange in a head-to-tail fashion, embracing all Domain I and Domain II α-helices at the inner face of the ring. The diameter of the inner ring is approximately 30 Å.

Trimerization of *Asfv*PCNA is mediated by various types of interactions. As depicted in Fig. 1D, the two strands β9 and β13 from the neighboring *Asfv*PCNA protomers reside next to each other, forming seven hydrogen bond (H-bond) interactions between their main chains. The side chains of α6 Lys193 and Tyr201 residues form H-bond interactions with the main chains of α3 Ser117 and Leu110 and Met114 residues, respectively (Fig. 1E). Like β9 and β13, the two α-helices are also arranged in an antiparallel orientation. In addition to direct H-bond interactions, *Asfv*PCNA trimerization is further stabilized by several water-mediated H-bond interactions (Fig. 1F).

### Conformational flexibility of *Asfv*PCNA

**Fig 2:**
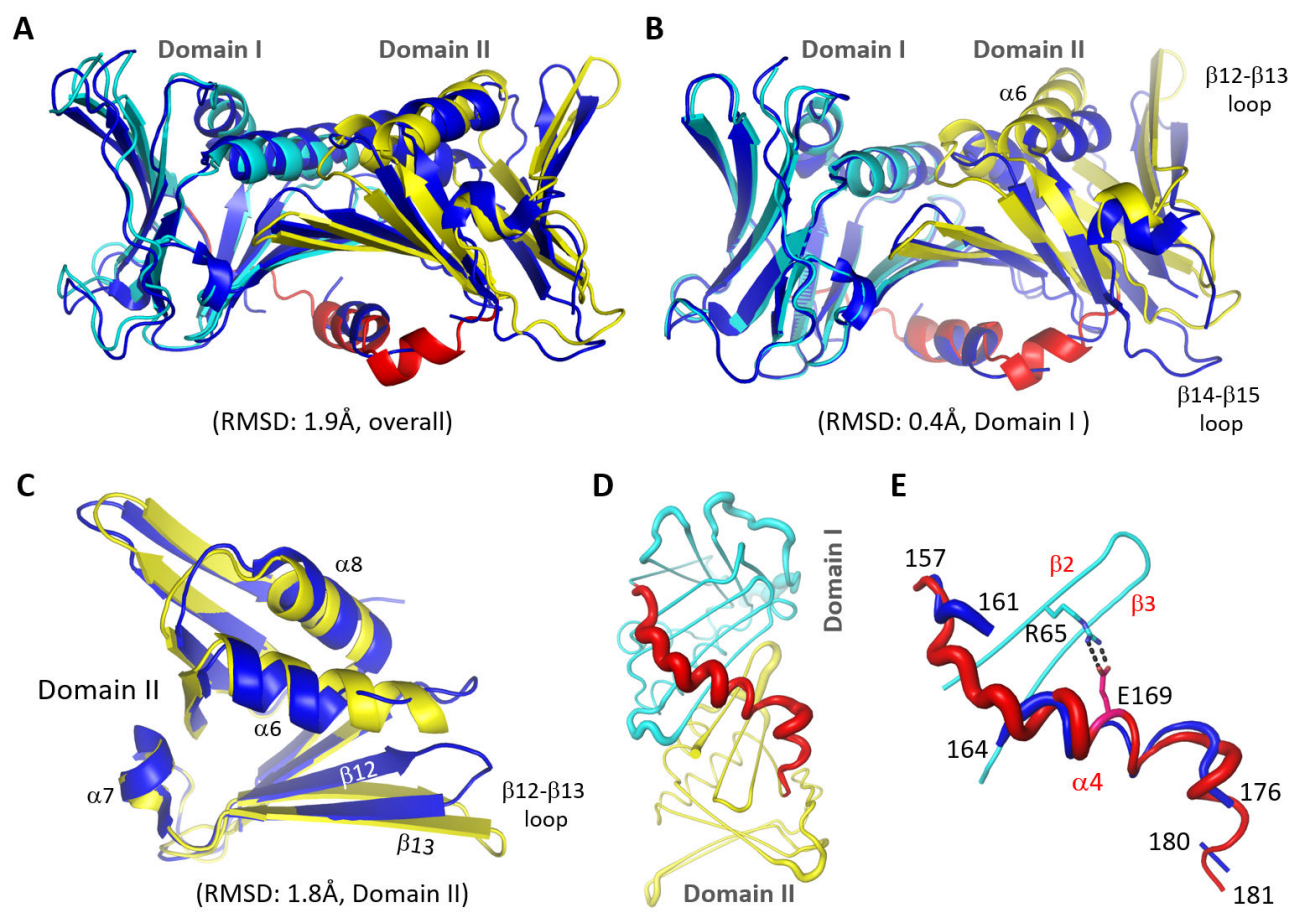
Comparison of Form I *Asfv*PCNA with the 7DRH structure. (**A**) Superposition of Form I *Asfv*PCNA with the 7DRH structure. (**B**) Structural superposition showing the orientational difference between Domain I and Domain II in Form I and 7DRH structures. (**C**) Superposition showing conformational changes in Domain II in Form I and 7DRH structures. (**D**) B-factor putty presentation of Form I *Asfv*PCNA structure. (**E**) Superposition of IDCL linkers in Form I and 7DRH structures. Domain I, Domain II, and IDCL of Form I structure are colored as in Fig. 1A, whereas 7DRH structure is colored in blue in all panels.

**Fig 3:**
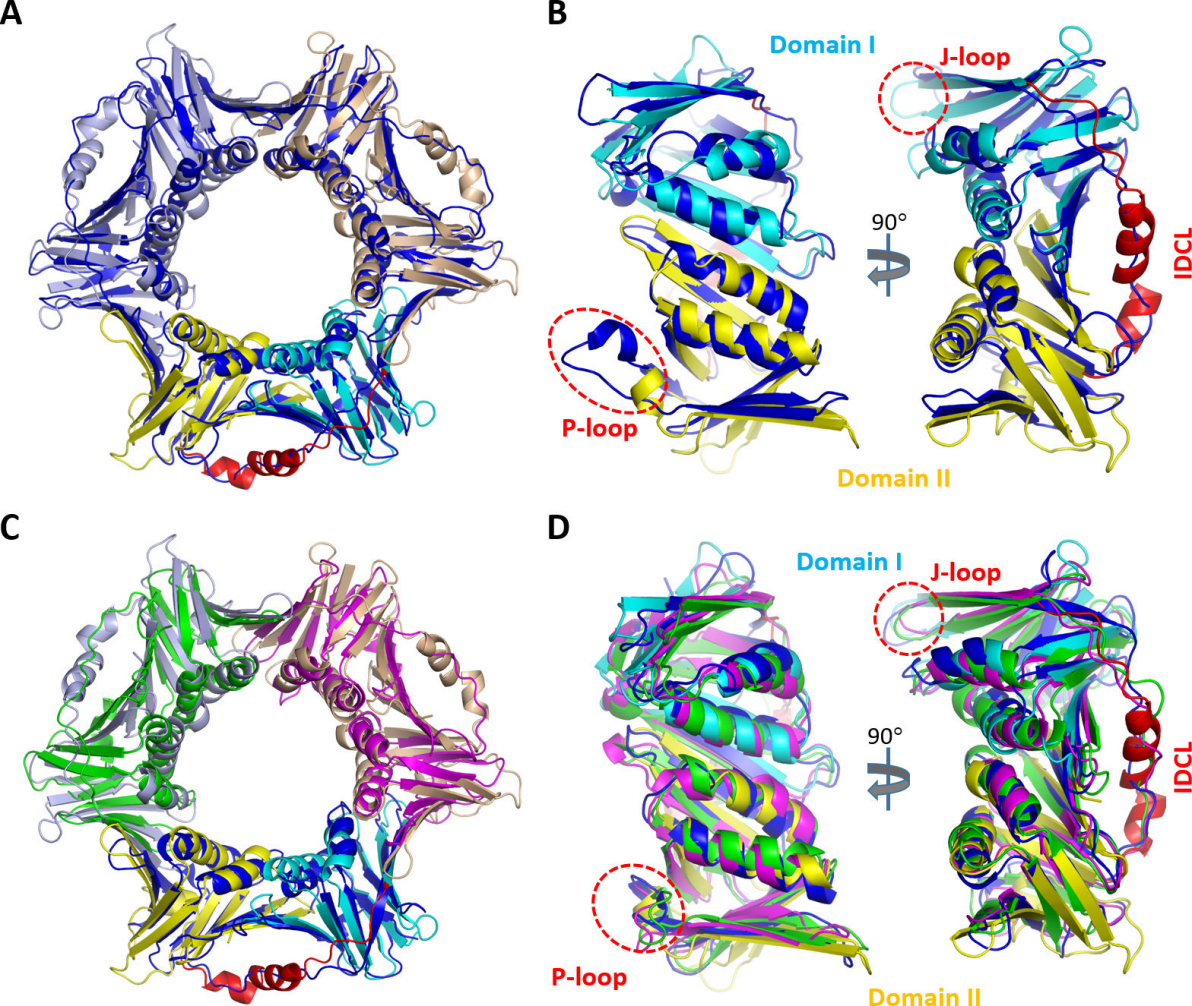
Comparison of *Asfv*PCNA with homologous PCNA proteins. (**A**) Superposition of *Asfv*PCNA with the *Sc*PCNA structure (PDB_ID: 3K4X), which is colored in blue. (**B**) Structural superposition showing the conformational differences between the protomers of *Asfv*PCNA and *Sc*PCNA. (**C**) Superposition of *Asfv*PCNA with the *Ss*PCNA structure (PDB_ID: 2HII). The three *Ss*PCNA protomers are colored in blue, green, and magenta, respectively. (**D**) Structural superposition showing the conformational differences between *Asfv*PCNA and *Ss*PCNA protomers. In panels A and C, the *Asfv*PCNA structure is colored as in Fig. 1A.

In addition to the two structures determined in this study, one additional *Asfv*PCNA structure was recently deposited in the protein data bank (PDB_ID: 7DRH). Although they possess identical amino acid sequences, the overall folding of the 7DRH structure is very different from that of the Form I or Form II *Asfv*PCNA structure; the RMSD value between them is close to 1.9 Å over 252 pairs of Cα atoms (Fig. 2A). The overall folding of Domain I is well conserved in all *Asfv*PCNA structures, supported by the low RMSD value (0.4 Å). In contrast to Domain I, the relative orientation and the detailed conformation of Domain II are very different in the *Asfv*PCNA structures (Fig. 2B and C). When superimposed on Domain I, there are more than 6 Å movement for the residues at the middle of Domain II β12–β13 and β14–β15 connecting loops (Fig. 3B). Compared to Form I structure, the α6 helix undergoes approximately 20° rotation in the 7DRH structure (Fig. 2B).

Large conformational differences were also observed for *Asfv*PCNA Domain IIs when they were directly superimposed on each other (Fig. 2C); the RMSD value between the Domain IIs is 1.8 Å. Instead of forming a continuous helix (α6) with residues _188_SKQLQQTF_195_, the residues _196_SDLSN_200_ are unfolded into loops in the 7DRH structure. The conformations of β12 N-terminus and β13 C-terminus are well conserved, but the N-terminus of β13 is tilted in the 7DRH structure. More dramatically, conformational changes occur in the C-terminus of β12, pointing the β12–β13 connecting loop to a completely different direction.

Compared to Domain I and Domain II, IDCL of *Asfv*PCNA is more flexible, as indicated by the higher B-factors (Fig. 2D). Not surprise, the IDCL also showed obvious conformational differences in the 7DRH and Form I *Asfv*PCNA structures (Fig. 2E). In the 7DRH structure, the regions _162_DM_163_ and _177_LKN_179_ are completely disordered. However, the conformations of the α4 helix are well conserved in the two structures, likely due to the strong electrostatic interactions between the side chains of IDCL Glu169 and Arg65 of the β2 strand (Fig. 2E; Fig. S3B).

Different from Form I and Form II structures, the 7DRH structure belongs to I4 space group, which contains one *Asfv*PCNA molecule per asymmetric unit. Instead of trimer, the 7DRH structure forms a square-like tetramer with a diameter of 50 Å for the inner ring in the crystal lattice (Fig. S5A). Structural comparison showed that the intermolecular interactions mediated by β9 and β13 are conserved in all the *Asfv*PCNA structures. However, the interactions between α3 and α6 are not observed in the 7DRH structure, likely due to the rotation of α6 (Fig. S5B). In addition to the conformational flexibility of the *Asfv*PCNA protomer, molecular packing and crystallization condition may also contribute to the formation of trimeric or tetrameric *Asfv*PCNA structure.

### Comparison of *Asfv*PCNA with other PCNA proteins

The structures of many eukaryotic PCNAs have been reported, including *Sc*PCNA, *Nc*PCNA, *Arabidopsis thaliana* (*At*) PCNA, *Drosophila melanogaster* (Dm) PCNA, and *Hs*PCNA, which all exist as homotrimers (50 - 54). The structures of many archaeal PCNAs have also been reported, such as *Ss*PCNA, *Archaeoglobus fulgidus* (*Af*) PCNA, *Haloferax volcanii* (*Hv*), *Pyrococcus abyssi* (*Pa*) PCNA, and *Thermococcus gammatolerans* (*Tg*) PCNA. *Af*PCNA, *Hv*PCNA, *Pa*PCNA, and *Tg*PCNA assemble into homotrimer (27, 55 - 57), whereas *Ss*PCNA is a heterotrimer (58). Compared to these archaeal and eukaryotic PCNAs, the length of *Asfv*PCNA is longer. *Asfv*PCNA contains one extra nonstructural loop (residues 1–30) at its N-terminus, the function of this extra loop is unclear at present.

Compared to the 7DRH structure, our *Asfv*PCNA structure is more similar to the reported PCNA structures in overall folding and assembly. As depicted in Fig. 3; Fig. S6 and S7, our *Asfv*PCNA structure can superimpose well with the eukaryotic and archaeal PCNA structures; the diameters of their inner rings are comparable. The structural similarity suggests that *Asfv*PCNA may be able to function as trimer. Previous studies showed that the sequences and conformations of PCNAs could be different at various regions, especially the J-loop, IDCL, and P-loop regions. While J-loop and IDCL were proposed to play important roles in species-specific partner recognition (54), the P-loop plays a regulatory role through post-translational modifications (59, 60).

The overall folding is conserved in *Asfv*PCNA and homologous PCNA proteins, but the sequence of *Asfv*PCNA is quite unique. Structure-based alignment (Fig. S8) showed that *Asfv*PCNA IDCL is much longer and has no clear sequence similarity with IDCLs of other PCNAs. IDCLs exhibit loop-like conformation in all reported PCNA structures (Fig. 3; Fig. S6 and S7), whereas it forms two short α-helices (α4 and α5) in the *Asfv*PCNA structure. The length of the P-loop of *Asfv*PCNA is similar to that of *Af*PCNA and *Hv*PCNA, but is shorter than other PCNA proteins, especially the eukaryotic PCNAs. *Asfv*PCNA P-loop contains four residues (_234_SSNK_237_), forming one short helix (α7). The P-loops also contain one short helix in the *Sc*PCNA (Fig. 3B), *Nc*PCNA, and *At*PCNA structures (Fig. S6D), but locates at a different position.

*Asfv*PCNA J-loop (_143_DFDIDK_148_) is composed of six residues, which is identical to *Sc*PCNA, *Nc*PCNA, *At*PCNA, *Dm*PCNA, and *Hs*PCNA (Fig. S8). The fifth and sixth residues are relatively conserved, but the other four residues are variable. Compared to these eukaryotic PCNA proteins, *Asfv*PCNA J-loop is more negative in charge; out of the six residues, three are Asp residues (Asp143, Asp145, and Asp147). The length of *Asfv*PCNA J-loop is similar to that of *Ss*PCNA, but is longer than *Af*PCNA, *Hv*PCNA, *Pa*PCNA, and *Tg*PCNA. No sequence similarity could be observed between the J-loops of *Asfv*PCNA and the archaeal PCNA proteins (Fig. S8). Previous studies showed wild-type *Nc*PCNA is not functional in *S. cerevisiae*, but introducing *Nc*PCNA with *Sc*PCNA J-loop can rescue the growth of *S. cerevisiae pol30Δ* PCNA deletion strain, indicating the important role of J-loop. In the future, it is worth investigating whether the J-loop of *Asfv*PCNA also plays a certain functional role in ASFV.

Like IDCL, J-loop, and P-loop, the C-terminal tail (C-tail) is also important for the function of many PCNAs (7, 61, 62). The sequences of the C-tail are relatively conserved in eukaryotic and archaeal PCNAs (Fig. S8). As observed in many PCNA structures, the first four residues of the C-tail are ordered, but the following residues are disordered, indicating their high flexibility in conformation. The C-tails of eukaryotic and archaeal PCNAs all contain a number of negatively charged residues (either Asp or Glu) at or near the very C-termini. However, the C-tail (_297_LNNTI_301_) of *Asfv*PCNA has no sequence similarity with either the eukaryotic or the archaeal PCNAs (Fig. S8). Although it is ordered in the 7DRH structure, the four residues (_298_NNTI_301_) are all disordered in the Form I and Form II *Asfv*PCNA structures, suggesting that *Asfv*PCNA C-tail is very flexible and can undergo large conformational changes in solution.

### Comparison of *Asfv*PCNA with homologous proteins from other viruses

**Fig 4:**
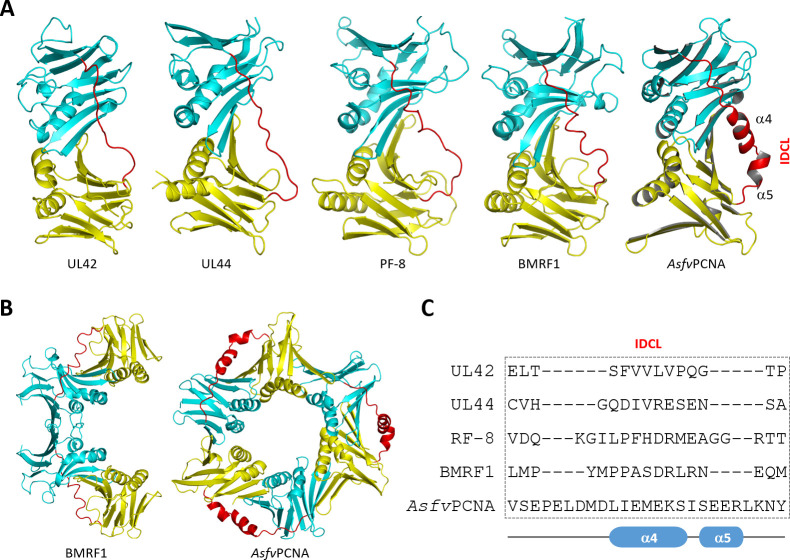
Comparison of *Asfv*PCNA with sliding clamp structural homologs from other viruses. (**A**) Comparison of the protomers of UL42 (PDB_ID: 1DML), UL44 (PDB_ID: 1T6L), PF-8 (PDB_ID: 3HSL), BMRF1 (PDB_ID: 2Z0L), and *Asfv*PCNA. (**B**) Comparison of the head-to-head dimer of BMRF1 and the head-to-tail trimer of *Asfv*PCNA. (**C**) Comparison of the IDCL sequence of *Asfv*PCNA and the viral sliding clamps. Domain I, Domain II, and IDCL linker are colored in cyan, yellow, and red, respectively.

**Fig 5:**
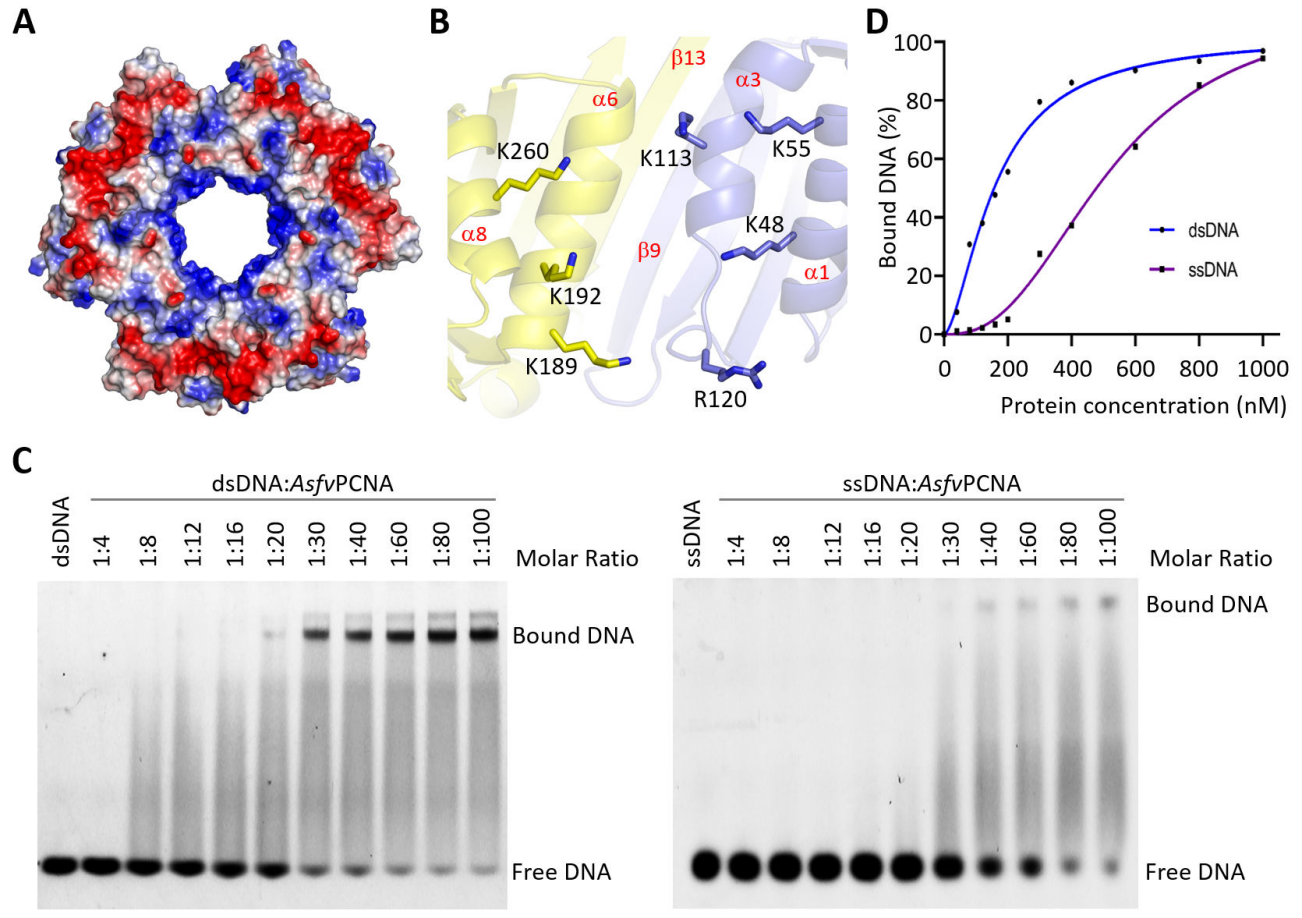
Verification of DNA binding by *Asfv*PCNA. (**A**) Surface electrostatic potential of *Asfv*PCNA trimer. The negative and positive residues are colored in red and blue, respectively. (**B**) Conformation of positively charged Lys and Arg residues from Domain I and Domain II of two neighboring protomers, which are colored in light blue and yellow, respectively. (**C**) *In vitro* EMSA assay showing DNA binding by *Asfv*PCNA. (**D**) Comparison of the dsDNA- and single-stranded DNA (ssDNA)-binding curves of *Asfv*PCNA.

In addition to ASFV, PCNA homologous proteins are also present in many other viruses. Although the homologous protein structures for many viruses are still unavailable, the structures of HSV-1 virus UL42, HCMV virus UL44, KSHV virus PF-8, and Epstein–Barr virus BMRF1 have been reported (33 - 63 - 63). As depicted in Fig. 4A, UL42, UL44, PF-8, and BMRF1 are composed of two domains: Domain I and Domain II, which are located at the N- and C-termini, respectively; the overall folding and the relative orientations between the two domains are similar to that of *Asfv*PCNA. However, the functional states of these viral proteins are different from *Asfv*PCNA (Fig. 4B). Instead of trimer, UL42 exists as monomer. UL44, PF-8, and BMRF1 all function as dimers with a C-shaped conformation; the two protomers are arranged in a head-to-head manner. In the *Asfv*PCNA structure, the three protomers are arranged in a head-to-tail manner: Domain I of one protomer interacts with Domain II of the neighboring protomer. Like *Asfv*PCNA, the Domain I and Domain II of UL42, UL44, PF-8, and BMRF1 are also connected by an IDCL linker (Fig. 4A). As confirmed by their crystal structures, the IDCL linkers play a critical role in partner recognition by these viral sliding clamp proteins. The IDCL linker of *Asfv*PCNA is longer and shares no sequence similarity with those of UL42, UL44, PF-8, and BMRF1 (Fig. 4C), which may lead to the different IDCL conformations observed in the structures. The predicted DNA-interacting surfaces of UL42 and UL44 contain several Lys and Arg residues (Fig. S9C and D), but their detailed distributions and conformations are very different from those of *Asfv*PCNA, *Sc*PCNA, and *Hs*PCNA (Fig. 5B; Fig. S9C and D).

### *Asfv*PCNA possesses high dsDNA-binding affinity

Like other eukaryotic and archaeal PCNA structures, the ring-shaped *Asfv*PCNA structure can be divided into four parts: the outer surface, the inner surface, the top side, and the bottom side. The top side is also termed the C side because the C-termini of the PCNA protomers all protrude from this side. As depicted in Fig. 5A, the electrostatic potential of *Asfv*PCNA is unevenly distributed. The C side is highly negative at the external region, due to the presence of Glu160, Asp162, Glu164, and Glu167 of IDCL and Glu276 and Glu277 of Domain II. The internal region of the C side is mainly composed of hydrophobic and positively charged residues, such as Lys222 and Lys255.

Compared to other parts, the inner surface of *Asfv*PCNA is more positive in charge (Fig. 5A). The side chains of Lys48, Lys55, Lys113, and Arg120 of Domain I and Lys189, Lys192, and Lys260 of Domain II all point toward the inner channel. Instead of the same protomer, these positively charged residues from the neighboring protomers are close to each other in space (Fig. 5B). *Hs*PCNA and *Sc*PCNA possess similar number of positively charged residues (Lys or Arg) at the inner surface of rings. The locations of these residues are conserved in *Hs*PCNA and *Sc*PCNA (Fig. S9A and B). However, out of the seven positively charged residues of *Asfv*PCNA, only Lys55 and Lys113 are relatively conserved in position.

PCNAs can interact with various types of partner proteins, including polymerase, ligase, and Fen1 endonuclease that all involve in DNA replication and/or repair pathway. To enhance the catalytic activity of the partner protein, PCNA should be able to form a stable complex with the partner protein and slide freely along the substrate DNA. The structures of *Pa*PolD-PCNA-DNA (Fig. S10A) and *Sc*Polδ-PCNA-DNA (Fig. S10B) have been reported (26, 27), showing that DNA was held by the polymerase and threaded through the central channel of the PCNA ring. The orientation of the DNA is roughly perpendicular to the plane of the ring. Similar orientations were also observed for DNA and β clamp in the *Ec*PolIII-β clamp-DNA complex structure (Fig. S10C) (64).

We could not obtain any *Asfv*PCNA crystal in the absence of dsDNA, whereas Form I and Form II *Asfv*PCNA crystals readily grew when 10 bp dsDNA (5′-CCCATCGTAT-3′; 5′-ATACGATGGG-3′) is present in the sample. In the structures, extra electron density maps could be observed in the central channel (Fig. S11A), suggesting the existence of dsDNA. The existence of dsDNA could be further supported by staining the crystals using GelRed, a nucleic acid-specific stain (Fig. S11B and C). Likely, due to the dynamic binding or sliding of the DNA, the electron density is diffused, and no DNA was included in the final structures. Weak electron density was also observed for DNAs in some previously reported PCNA/DNA complex structures (50). To further confirm the DNA-binding ability of *Asfv*PCNA, we performed *in vitro* EMSA assays (Fig. 5C). Compared to single-stranded (ssDNA), the dsDNA-binding affinity of *Asfv*PCNA is higher (Fig. 5D). The calculated dissociation values (*K*_d_) are 0.16 µM and 0.52 µM for the dsDNA and ssDNA, respectively. Taken together, these observations suggested that *Asfv*PCNA possesses high dsDNA-binding affinity, and the dsDNA is likely bound in the central channel with an orientation similar to those in the reported polymerase ternary complex structures (Fig. S10).

### *Asfv*PCNA modestly enhances the ligation activity of *Asfv*LIG

ASFV is one of the most complex dsDNA viruses known to date. In addition to PCNA, the genome of ASFV also encodes many other proteins involved in DNA replication and repair, such as replicative DNA polymerase, topoisomerase II, and ligase (65). Maybe due to the difficulties in expression and purification, the structures of ASFV replicative polymerase and topoisomerase II remain elusive. The structure of ASFV ligase (*Asfv*LIG) was determined by our group previously (48). Like *Hs*LIG (Fig. S12A) and all other homologous proteins, *Asfv*LIG is composed of three domains: the DNA-binding domain (DBD) at the N-terminus, the adenylation domain (AD) in the center, and the OB-fold domain (OB) at the C-terminus (Fig. S12B).

**Fig 6:**
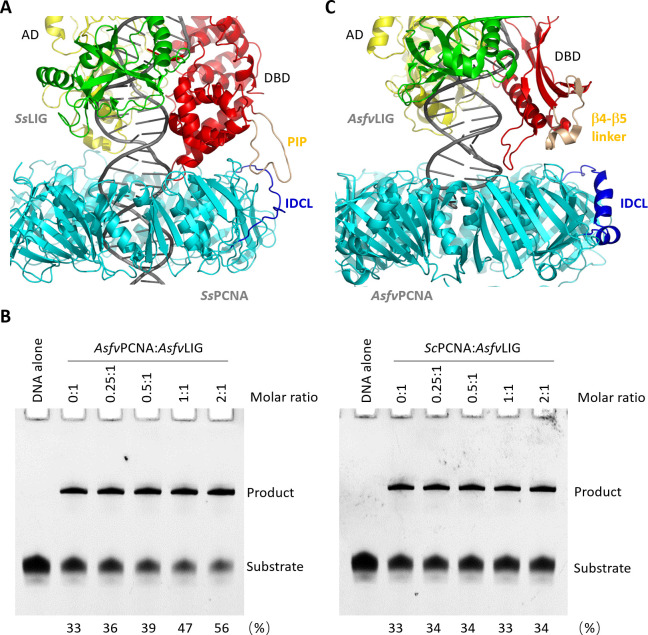
*Asfv*PCNA facilitates DNA ligation by *Asfv*LIG. (**A**) Cryo-EM structure of archaeal *Ss*PCNA-LIG-DNA ternary complex. The interacting IDCL of *Ss*PCNA and PIP of *Ss*LIG are colored in blue and wheat, respectively. (**B**) *In vitro* DNA ligation assays showing the impacts of *Asfv*PCNA and *Sc*PCNA on the catalytic activity of *Asfv*LIG. The percentage of the product is given at the bottom of the image. (**C**) Superposition of *Asfv*LIG/dsDNA complex (PDB_ID: 6IMK) and *Asfv*PCNA onto the *Ss*PCNA-LIG-DNA structure. For clarity, the *Ss*PCNA-LIG-DNA structure was omitted.

Interestingly, although they share similar domain architectures, the sequence similarities between *Asfv*LIG and the homologous proteins are very low, especially at the DBD domain region. The DBD of canonical ligase is approximately 280 amino acids in size and is of α-fold in nature. The DBD domain of *Asfv*LIG (amino acids 1–120) is much shorter; in addition to α-helices, it also contains several β-strands. The *Ss*PCNA-*Ss*LIG-DNA ternary complex structure (PDB_ID: 7RPX) was recently reported (28), showing that DNA was bound in the central channel of *Ss*PCNA (Fig. 6A). The IDCL loop of *Ss*PCNA plays the most critical roles in the ternary complex assembly; it directly recognizes the PCNA-interacting peptide (PIP) motif of *Ss*LIG, which locates in the DBD domain.

As aforementioned, both the IDCL linker of *Asfv*PCNA (Fig. 3 and 4) and the DBD domain of *Asfv*LIG (Fig. S11B) are unique, which raises the question whether *Asfv*PCNA can enhance the ligation activity of *Asfv*LIG. To this end, we performed *in vitro* ligation assays. As depicted in Fig. 6B (left panel), *Asfv*LIG is active in the absence of *Asfv*PCNA; however, the activity is relatively weak. Addition of 0.5 and 1.0 molar ratios of *Asfv*PCNA (trimer) can slightly enhance the ligation activity. Addition of 2 molar ratio of *Asfv*PCNA in the reaction system leads to an approximately twofold enhancement in DNA ligation catalyzed by *Asfv*LIG. No further enhancement is observed when more *Asfv*PCNA is included in the reaction system (Fig. S13). To test whether the enhancement by *Asfv*PCNA is specific, we purified the *Sc*PCNA protein (Fig. S14) and performed ligation assays under the same condition. In contrast to *Asfv*PCNA, the addition of *Sc*PCNA has no impact on DNA ligation catalyzed by *Asfv*LIG (Fig. 6B, right panel). Like *Sc*PCNA, the DNA-binding affinities of many other archaeal and eukaryotic PCNAs are also weak, whereas these PCNA proteins can significantly enhance the catalytic activities of their partner proteins. Instead of *Asfv*PCNA-DNA binding, we believe that the potential *Asfv*PCNA-*Asfv*LIG interaction may play a more significant role in the enhancement of the catalytic activity of *Asfv*LIG.

## DISCUSSION

Here we report the structure and functional studies of *Asfv*PCNA protein. Our structures showed that *Asfv*PCNA can assemble into homotrimer, which is similar to that adopted by eukaryotic and archaeal PCNAs, including *Hs*PCNA, *Sc*PCNA, *Ss*PCNA, *Tg*PCNA, and so on. Interestingly, although they all belong to the DNA sliding clamp family, *Asfv*PCNA shares no clear sequence similarity with the homologous proteins. As a result, *Asfv*PCNA adopts unique conformations at several regions (Fig. 3; Fig. S6 to S8), such as the J-loop, the IDCL linker, the P-loop, and the C-tail. It is of note that one trimeric *Asfv*PCNA structure was recently reported by Wu and coworkers, showing that the N-terminal extension of *Asfv*PCNA is also unique and plays an important role in the stabilization of *Asfv*PCNA homotrimer in solution (66).

Compared to eukaryotic and archaeal PCNAs, the IDCL linker of *Asfv*PCNA is much longer and contains two short α-helices (α4 and α5) near the C-terminus (Fig. 1A). Via interacting with the PIP-motif (PCNA interaction peptide) of the partner proteins, IDCL plays the most critical role in partner recognition by PCNA. The canonical PIP-motif is composed of Q-xx-ψ-x-x-θ-θ, in which x represents any residue, ψ and θ are hydrophobic (*e.g.*, M, L, and I) and aromatic (e.g. F and Y) residues, respectively (67, 68). By exploiting a random peptide display library, a novel PIP-motif with a sequence of K-A-(A/L/I)-(A/L/Q)-x-x-(L/V) was discovered. This novel motif was also termed the KA-motif and has been found in several PCNA partner proteins (69). Although both *Asfv*PCNA and *Asfv*LIG show obvious conformational differences from their homologous proteins (Fig. 3; Fig. S11), our *in vitro* ligation assay results (Fig. 6B) confirmed that *Asfv*PCNA can modestly enhance the ligation activity of *Asfv*LIG. Sequence analysis did not identify any canonical PIP-like or KA-like motif in *Asfv*LIG, but structural superposition suggested that the β4–β5 linker of *Asfv*LIG DBD is likely involved in interaction with *Asfv*PCNA IDCL (Fig. 6C). The conformation of *Asfv*LIG β4–β5 linker is very different from the canonical PIP- or KA-motif. We speculated that *Asfv*LIG β4–β5 linker and/or *Asfv*PCNA IDCL will undergo certain conformational changes to form stable interactions.

To enhance the catalytic activities of polymerase, ligase, and nuclease, PCNA needs to encircle and freely slide along the substrate DNA. However, eukaryotic and archaeal PCNAs, such as *Hs*PCNA, *Sc*PCNA, and *Ss*PCNA, exist as stable trimer with a closed ring-shaped overall structure. In all known cellular life, PCNA loading onto DNA requires the help of the RFC factor, which is composed of five protein molecules. A very recent study showed that PCNA loading is a coordinated and stepwise process, including ATP binding by RFC, PCNA-RFC complex formation, large-scale expansion of RFC, PCNA opening, DNA binding, PCNA closure, ATP hydrolysis, and RFC ejection (70). Unlike the eukaryotic and archaeal PCNAs, our *in vitro* assays showed that *Asfv*PCNA has strong dsDNA-binding ability (Fig. 5C and D). The *K*_d_ value of *Asfv*PCNA is comparable to that of the viral sliding clamp proteins, such as UL42 and UL44, which do not require RFC for DNA binding. In addition to positively charged Lys and Arg residues located at the inner surface, the recent study suggested that the N-terminal extension also plays a certain role in DNA binding by *Asfv*PCNA (66). As demonstrated by our structures (Fig. 1C) and the 7DRH structure (Fig. S4), *Asfv*PCNA can exist as either trimer or tetramer with a central channel. At present, it is unclear whether both trimeric and tetrameric *Asfv*PCNAs are functional and whether RFC or a similar factor is required for opening and loading *Asfv*PCNAs onto target dsDNA *in vivo*.

Owing to their important biological functions, PCNAs have been considered a potential antibacterial and anticancer drug target (31). Various types of PCNA inhibitors have been identified, including natural products, small molecule inhibitors, and peptides. Peptide inhibitors generally mimic the PIP-motif in sequence, and they share similar PCNA-binding mode with many known PCNA partners, such as Fen-1 nuclease. Peptide inhibitors can also be derived from other PCNA-interacting motif, such as the AlkB homolog 2 PCNA-interacting motif (APIM). Compared to the canonical PIP-motif, the APIM motif is short; it is only composed of five residues, (K/R)(Y/Y/W)(L/I/V/A)(L/I/V/A)(K/R) (71). Although they are very different in sequence, these peptide inhibitors normally bind to the PIP-binding pocket and form H-bond and hydrophobic interactions with the IDCL linker of PCNA, blocking the binding of PCNA partners. Like griselimycins (32), nonsteroidal anti-inflammatory drugs, 3,3′,5-triiodothyronine (T3), and some other small molecule inhibitors also target the PIP-binding pocket, inhibiting partner protein binding and interacting with the IDCL linkers of the sliding clamp proteins (72, 73). The sequence and conformation of *Asfv*PCNA IDCL is very unique (Fig. 3 and 4), which may allow the development of *Asfv*PCNA-specific peptide inhibitors.

In addition to the PIP-binding pocket, peptide and small molecule inhibitors can also bind to other regions of PCNA. For example, the small molecule T2AA (an analog of triiodothyronine) can bind at the interface between the trimer subunits, block PCNA ubiquitination, and inhibit partner interaction (73). It was reported that T2AA can regulate how cells respond to DNA damage; cells treated with T2AA are more sensitive to the cancer therapeutic cisplatin and have a lower survival rate. Binding of small molecule and canonical PIP-like or APIM-like peptide inhibitors usually does not alter the overall structures of PCNAs. In contrast, binding of some small proteins, such as Thermococcales inhibitor of PCNA (TIP), can lead to obvious conformational changes of PCNA (74). TIP alone is partially disordered, whereas it becomes structured upon PCNA binding. TIP can interact with both Domain I and Domain II, altering the relative orientation between them and preventing PCNA from functional trimer assembling. As revealed by the structural superposition, Domain I and Domain II of *Asfv*PCNA can undergo large conformational changes (Fig. 2A and B). Small molecule and peptide that block oligomerization or fix the structure in a nonfunctional state will certainly disrupt the function of *Asfv*PCNA.

ASFV is one of the most complex DNA viruses known to date, its genome encodes more than 160 proteins. We previously reported the crystal structures of *Asfv*AP, *Asfv*PolX, and *Asfv*LIG proteins, which revealed many ASFV-specific structural features, such as the narrow abasic-binding site in *Asfv*AP (47), the novel 5′-P-binding pocket in the finger domain of *Asfv*PolX (49), and the unique DBD domain in the N-terminus of *Asfv*LIG (48). Here we show that *Asfv*PCNA is also very different from the homologous PCNA proteins in the J-loop, IDCL, the P-loop, and the C-tail regions (Fig. 3; Fig. S6 to S8). ASFV is the only member of the *Asfarviridae* family; the majority ASFV proteins share very low sequence similarity with the homologous proteins, which may explain the unique structural features possessed by *Asfv*AP, *Asfv*PolX, *Asfv*LIG, and *Asfv*PCNA. In the future, it is worth studying the structure and function of other ASFV proteins, such as the replicative DNA polymerase. Most likely, these proteins also contain some unique structural features that can be utilized as target for the development of ASFV-specific inhibitors and help combat the deadly virus.

## MATERIALS AND METHODS

### Protein expression and purification

The codon optimized *Asfv*PCNA gene (Table S1) and *Sc*PCNA (Table S2) gene were synthesized by GENEWIZ Co., Ltd, Suzhou, China. The target genes were amplified by polymerase chain reaction and subcloned into the pET-28a-Sumo vector between BamHI and XhoI restriction sites. The recombinant plasmids were transformed into *Escherichia coli* strain BL21 (DE3) and overexpressed. The cells were cultured at 37°C in 1 L Luria-Bertani medium containing 50 µg/mL of kanamycin. When the OD600 value reached 0.6–0.8, protein expression was induced by the addition of isopropyl β-D-1-thiogalacto-pyranoside at a final concentration of 0.05 mM. The induced cultures were then grown at 18°C for an additional 18–22 h.

Cell was harvested and resuspended in lysis buffer (20 mM Tris pH 7.0, 500 mM NaCl, and 25 mM imidazole) and homogenized with a low-temperature ultra-high pressure cell disrupter. The lysate was centrifuged at 25,000 × *g* for 30 min at 4°C to remove cell debris. The supernatant was loaded onto a HisTrap HP column equilibrated with lysis buffer. The fusion protein was eluted using elution buffer (20 mM Tris pH 7.0, 500 mM NaCl, and 500 mM imidazole) with a gradient. The fractions containing the desired fusion proteins were pooled and dialyzed against dialysis buffer (20 mM Tris pH 7.0 and 500 mM NaCl) at 4°C for 3 h, and Ulp1 protease was added to the sample during the dialysis process. The sample was again loaded onto the HisTrap HP column. The target proteins were collected, concentrated, and loaded onto a HiLoad 16/600 Superdex S200 column equilibrated with gel filtration buffer (20 mM Tris pH 7.0, 200 mM NaCl, and 2 mM DTT).

Selenomethionine-substituted *Asfv*PCNA protein was expressed in M9 medium supplemented with 60 mg/L Se-Met and purified using a procedure similar to that of the native protein. *Asfv*LIG protein was expressed and purified as previously described. Purity of all proteins was analyzed using a 15% SDS-PAGE gel, and the samples were stored at −80°C until use.

### Crystallization and data collection

Substrates DNA1 (5′-CCCATCGTAT-3′) and DNA2 (5′-ATACGATGGG-3′) were dissolved in gel filtration buffer. The DNA was annealed by heating to 95°C and slowly cooling to room temperature. Prior to crystallization, *Asfv*PCNA and the annealed dsDNA were mixed, the final concentration of *Asfv*PCNA is 15 mg/mL (corresponding to 0.14 mM *Asfv*PCNA trimer); the concentration of dsDNA is 0.21 mM. Initial crystallization conditions were screened by the sitting-drop vapor-diffusion method using commercial crystal screening kits at 18°C. The drop contained an equal volume (0.2 µL) of protein sample and reservoir solution and was equilibrated against 50 µL of reservoir solution in a 96-well format. Form I *Asfv*PCNA crystals were grown in the buffer composed of 0.1 M Tris pH 8.5 and 20% (w/v) PEG1000, whereas Form II crystals were grown in 0.1 M sodium chloride, 0.1 M BICINE pH 9.0, and 20% v/v PEG550 buffer.

All crystals were cryoprotected using their mother liquor supplemented with 25% glycerol and snap-frozen in liquid nitrogen. The diffraction data were collected at beamline BL18U1 at the Shanghai Synchrotron Radiation Facility. Data processing was carried out using the imosflm or HKL3000 program. The data collection and processing statistics are summarized in Table 1.

### Structure determination and refinement

Form I *Asfv*PCNA structure was solved by the single-wavelength anomalous diffraction method (75) with the Autosol program embedded in the Phenix suit (76). The initial model was built using the Autobuilt program and then refined against the diffraction data using the Refmac5 program of the CCP4 suite (77). The 2F_o_−F_c_ and F_o_−F_c_ electron density maps were used as guides for the building of the missing amino acids using COOT (78). Form II *Asfv*PCNA structure was solved by molecular replacement using the Form I structure as the search model with the phaser program of the CCP4 suite (79). The final refinement of both structures was performed using the phenix.refine program. The structural refinement statistics are also summarized in Table 1.

### Electrophoretic mobility shift assay

ssDNA (5′-FAM-CCCATCGTAT-3′) or dsDNA (5′-FAM-CCCATCGTAT-3′ and 5′-ATACGATGGG-3′) was mixed with different molar ration of *Asfv*PCNA in binding buffer (20 mM Tris pH 7.0 and 500 mM NaCl). The final concentrations are 10 nM for both ssDNA and dsDNA. Twenty microliters of reaction mixtures was incubated on ice for 1 h and then analyzed on 6% native PAGE gels with 0.5 × TBE (Tris-borate-EDTA) buffer. The gel was imaged using Typhoon FLA 9000. The intensities of the substrate bands were quantified by ImageQuant TL. The percentage of binding, for each protein concentration, was calculated. Data were then fitted to the equation *Y*  =  *B*_max_*X^h^/(*K*_d_^h^  +  *X*^h^) using nonlinear regression (curve fit) in GraphPad Prism. The dissociation constants (*K*_d_) were determined from the regression curve.

### DNA ligation assay

Substrate DNAs were assembled by mixing the template strand DNA (5′-ATGCCTTACCGTGCTTCGACAACGAGTCAAGCGCATCCCG-3′), downstream DNA (5′-PO4-GTCGAAGCACGGTAAGGCAT) and upstream DNA (FAM-5′-CGGGATGCGCTTGACTCGTT) in a molar ratio of 1:1:1. For catalysis assays, a 20-µL reaction system (composed of 10 mM MgCl_2_, 1 mM ATP, 500 nM DNA substrate, and 100 nM *Asfv*LIG and *Asfv*PCNA at different concentrations) was established in reaction buffer (200 mM NaCl, 20 mM Tris pH 8.0, and 2 mM DTT). The reactions were incubated at 37°C for 30 min and terminated by adding 20 µL termination buffer (90% formamide, 20 mM EDTA, 0.05% bromophenol blue, and 0.05% xylene blue) and boiling at 95°C for 5 min. The product was analyzed on 18% urea sequencing gel with TBE buffer. The gel was imaged using Typhoon FLA 9000.

## Data Availability

The atomic coordinates and structure factors have been deposited in the Protein Data Bank under accession code 7YPE and 7YPF for Form I and Form II *Asfv*PCNA structures, respectively.

## SUPPLEMENTAL MATERIAL

Supporting information jvi.00748-23-s0001.pdf This file contains 14 supporting figures and 2 supporting tables.

ASM does not own the copyrights to Supplemental Material that may be linked to, or accessed through, an article. The authors have granted ASM a non-exclusive, world-wide license to publish the Supplemental Material files. Please contact the corresponding author directly for reuse.
