# Supplementary material for: Structural and functional studies of PCNA from African swine fever virus
Source: J Virol. 2023 Aug 3;97(8):e00748-23. doi: 10.1128/jvi.00748-23 (PMC10506467; doi:10.1128/jvi.00748-23)
Supplement: Supporting information — This file contains 14 supporting figures and 2 supporting tables. [file jvi.00748-23-s0001.pdf]

Supporting Information

For

Structural and functional studies of PCNA from African Swine Fever Virus

Zhiwei Shao[1], Jie Yang[1], Yanqing Gao[1], Yixi Zhang[1], Xin Zhao[1], Qiyuan Shao[1], Weizhen Zhang[1], Chulei Cao[1], Hehua Liu[1], Jianhua Gan[1,*]

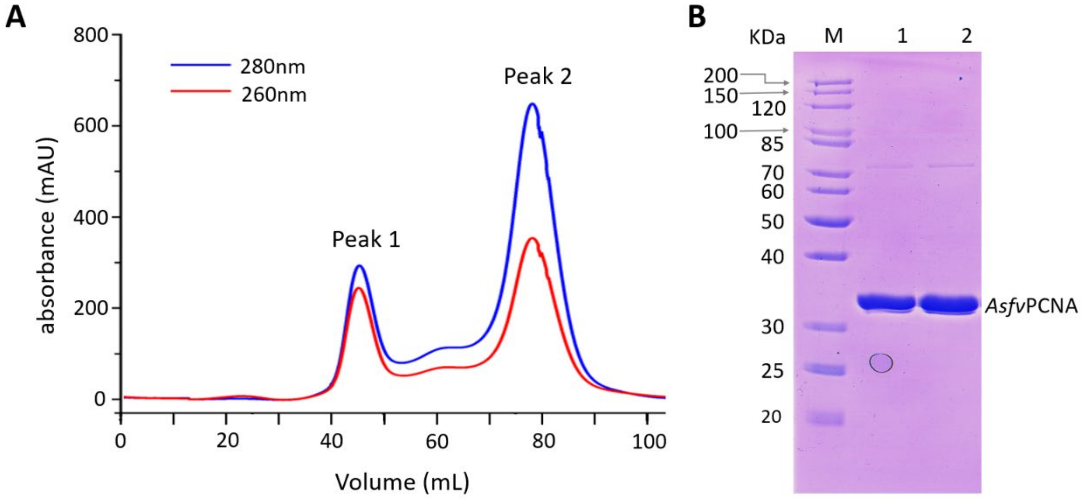

Fig. S1: Purification of *Asfv*PCNA. (**A**) Size-exclusion chromatographic analysis of *Asfv*PCNA. (**B**) SDS-PAGE gel analysis of *Asfv*PCNA. M, protein marker; 1 and 2, *Asfv*PCNA protein collected from peak 2 of the S200 16/600 size-exclusion column.

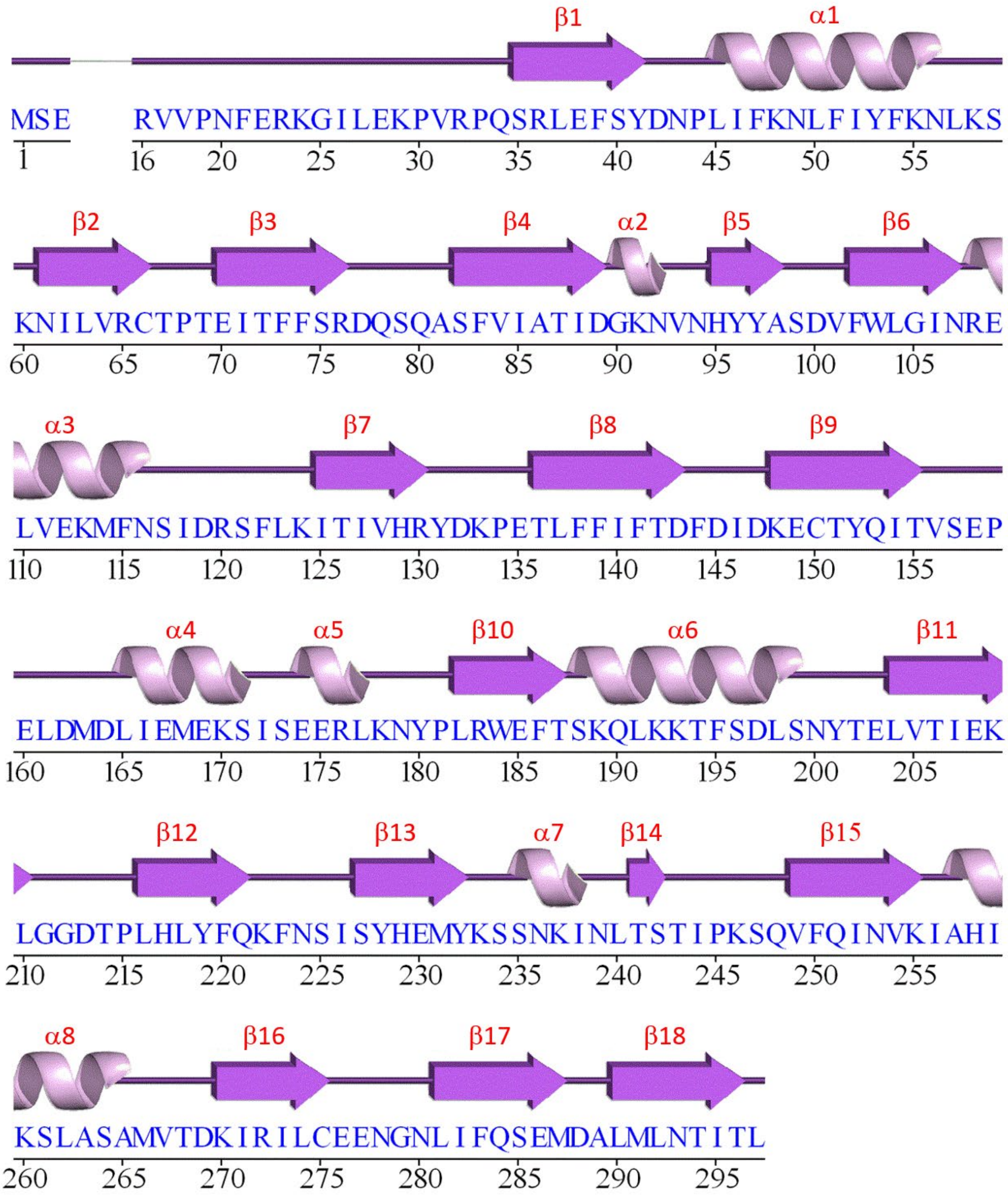

Fig. S2: Secondary structure of *Asfv*PCNA.

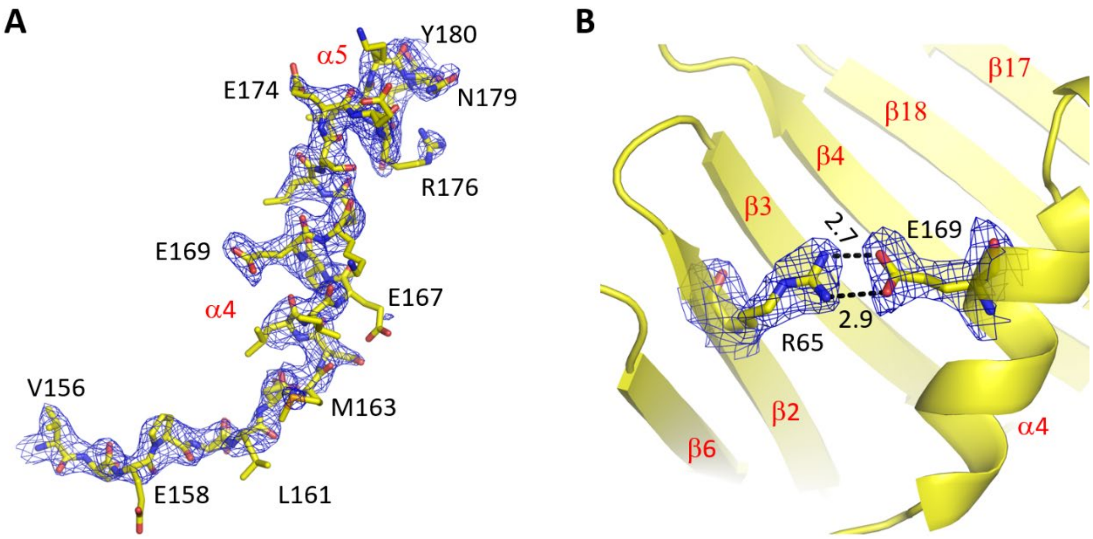

Fig. S3: Conformation of the IDCL linker of *Asfv*PCNA. (**A**) The detailed conformation of IDCL linker observed in Form I *Asfv*PCNA structure. (**B**) The detailed interactions between R65 of Domain I and E169 of IDCL in the Form I *Asfv*PCNA structure. The $2F_o$-F_c electron density maps are contoured at 1.2 σ level. The distances (Å) are shown in numbers.

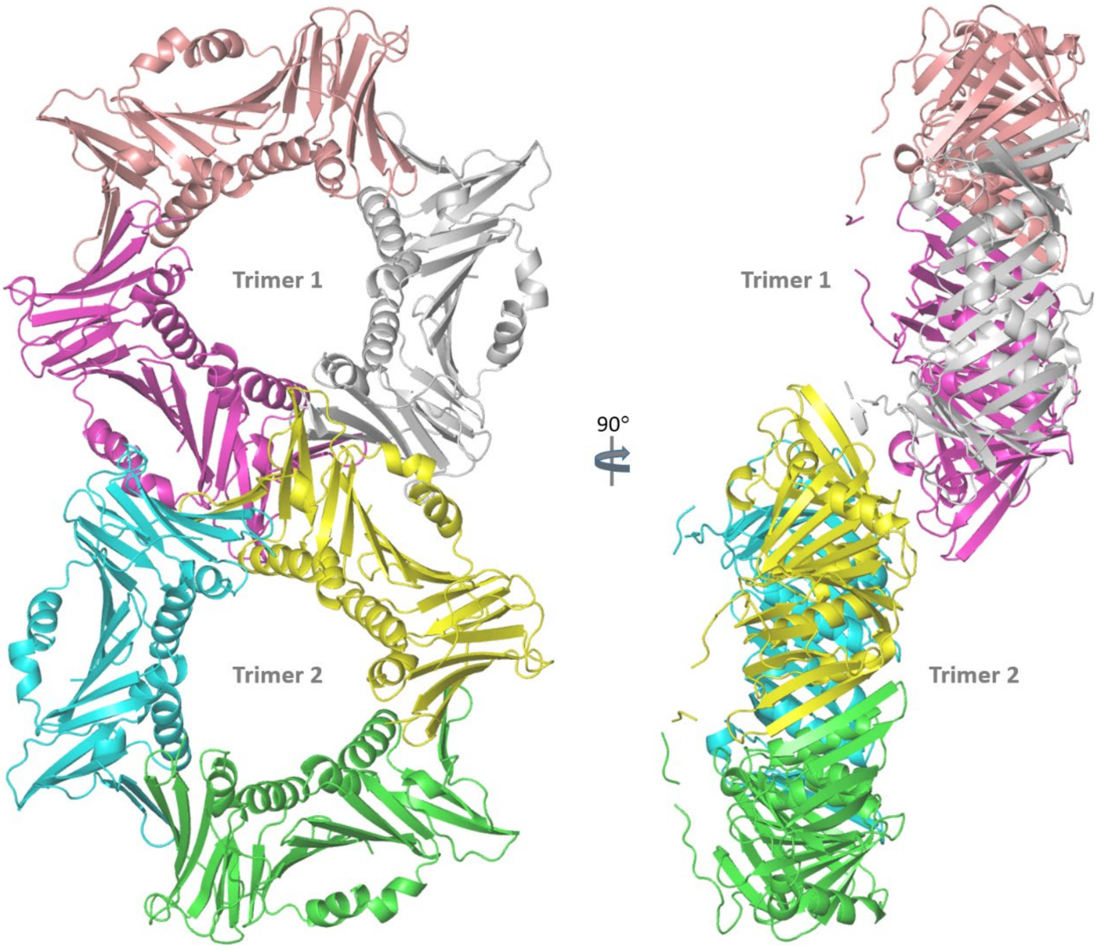

Fig. S4: Overall folding of *Asfv*PCNA in Form II structure. Per asymmetric unit contains six *Asfv*PCNA molecules, which assemble into two trimers, trimer 1 and trimer 2.

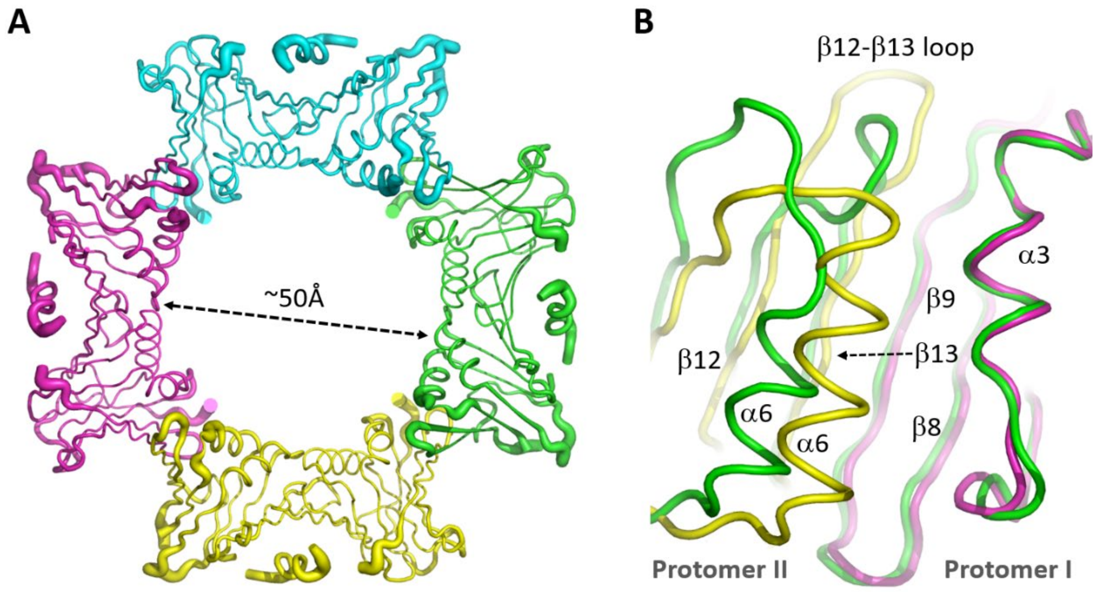

Fig. S5: Comparison between Form I *Asfv*PCNA and the 7DRH structures. (**A**) The overall folding of the tetrameric 7DRH structure. (**B**) Structural superposition showing the different protomer-interacting interfaces in Form I *Asfv*PCNA and the 7DRH structures. The two neighboring protomers are colored in green in the 7DRH structure, but in magenta and yellow in Form I *Asfv*PCNA structure.

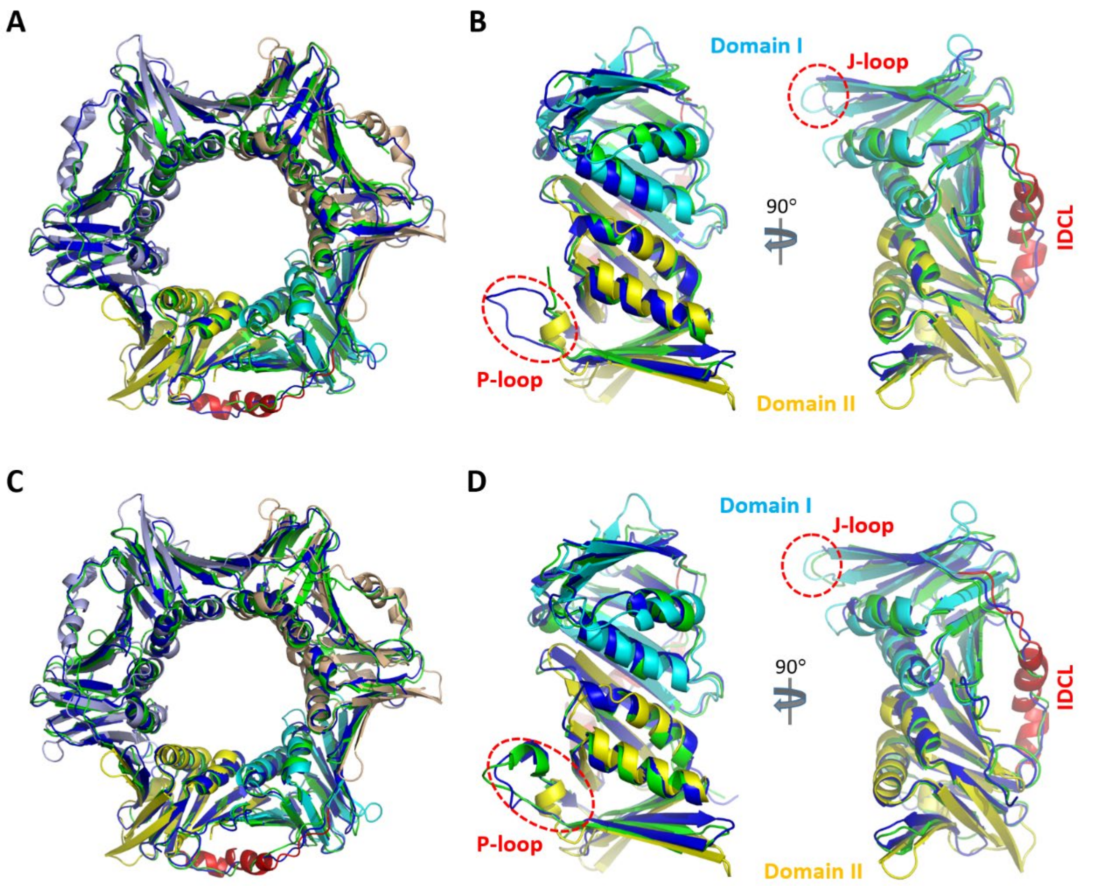

Fig. S6: Comparison between *Asfv*PCNA and eukaryotic PCNAs. (**A-B**) Comparison of *Asfv*PCNA with *Hs*PCNA (PDB_ID: 1VYM) and *Dm*PCNA (PDB_ID: 4KHI) structures, which are colored in blue and green, respectively. folding of the tetrameric 7DRH structure. (**C-D**) Comparison of *Asfv*PCNA with *At*PCNA (PDB_ID: 6O09) and *Nc*PCNA (PDB_ID: 7EP8) structures, which are colored in blue and green, respectively. *Asfv*PCNA structure is colored as in Fig. 1C.

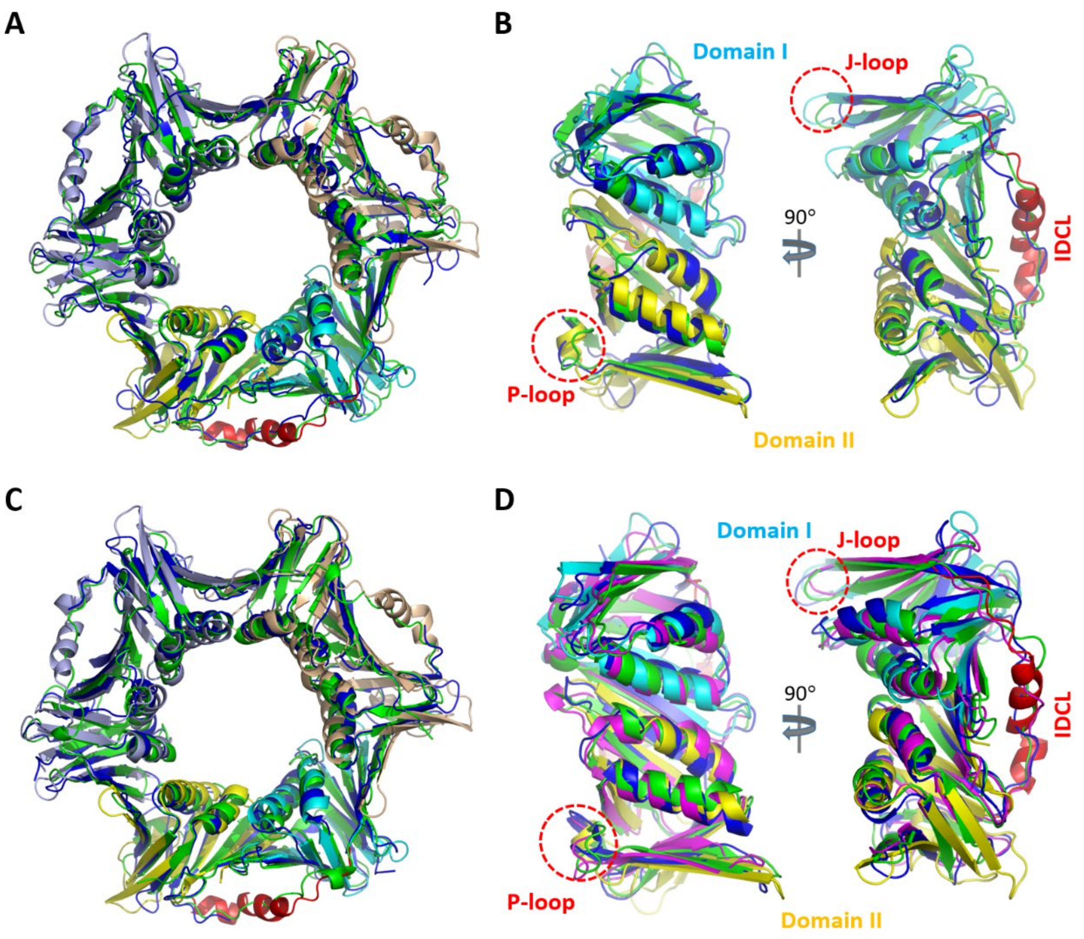

Fig. S7: Comparison between *Asfv*PCNA and archaeal PCNAs. (**A-B**) Comparison of *Asfv*PCNA with *Hv*PCNA (PDB_ID: 3HI8) and *Tg*PCNA (PDB_ID: 7N5K) structures, which are colored in blue and green, respectively. folding of the tetrameric 7DRH structure. (**C-D**) Comparison of *Asfv*PCNA with *Pa*PCNA (PDB_ID: 6T7X) and *Af*PCNA (PDB_ID: 1RWZ) structures, which are colored in blue and green, respectively. *Asfv*PCNA structure is colored as in Fig. 1C.

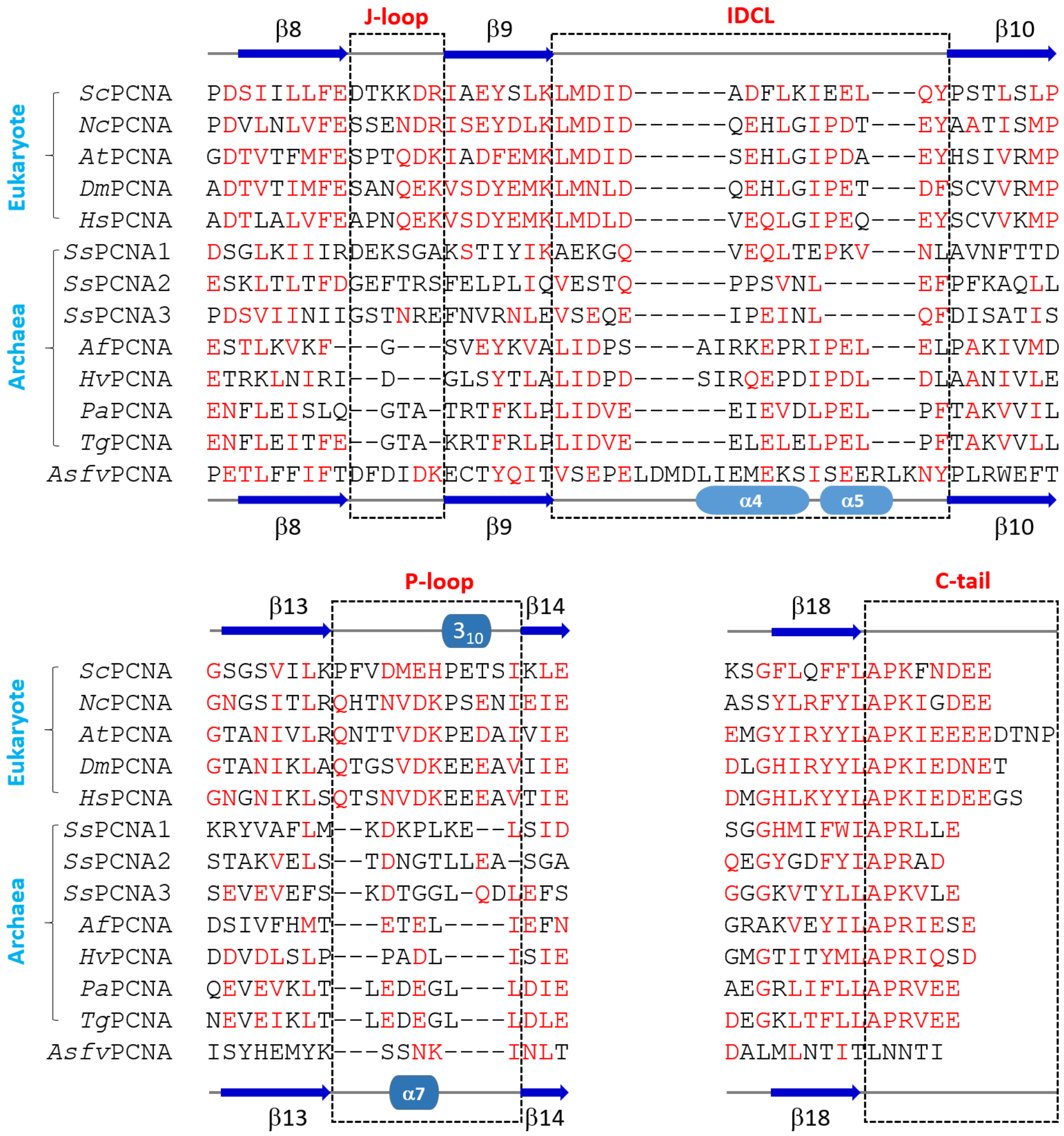

Fig. S8: Structure-based sequence alignment showing the difference in the J-loop, IDCL, P-loop and C-tail regions between *Asfv*PCNA and homologous PCNA proteins.

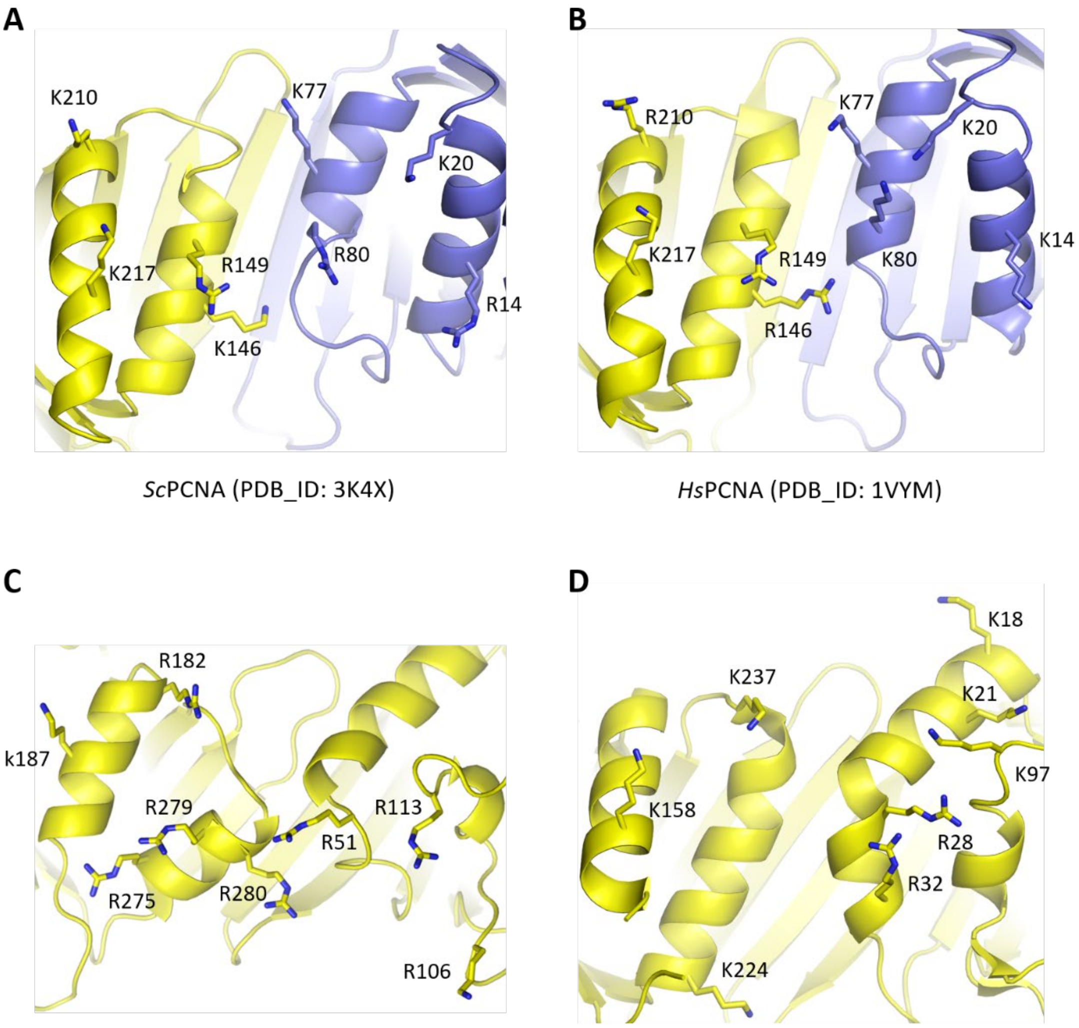

*Sc*PCNA (PDB_ID: 3K4X) *Hs*PCNA (PDB_ID: 1VYM)

UL42 (PDB_ID: 1DML) UL44 (PDB_ID: 1T6L)

Fig. S9: Distribution of positively charged residues on the surface of *Asfv*PCNA homologs. (A-B) Conformations of Lys and Arg residues located at the inner surfaces of *Sc*PCNA and *Hs*PCNA, respectively. Domain I and Domain II from two neighboring protomers are colored in light blue and yellow, respectively. **(C-D)** Conformations of Lys and Arg residues located at the surfaces of UL42 and UL44, respectively.

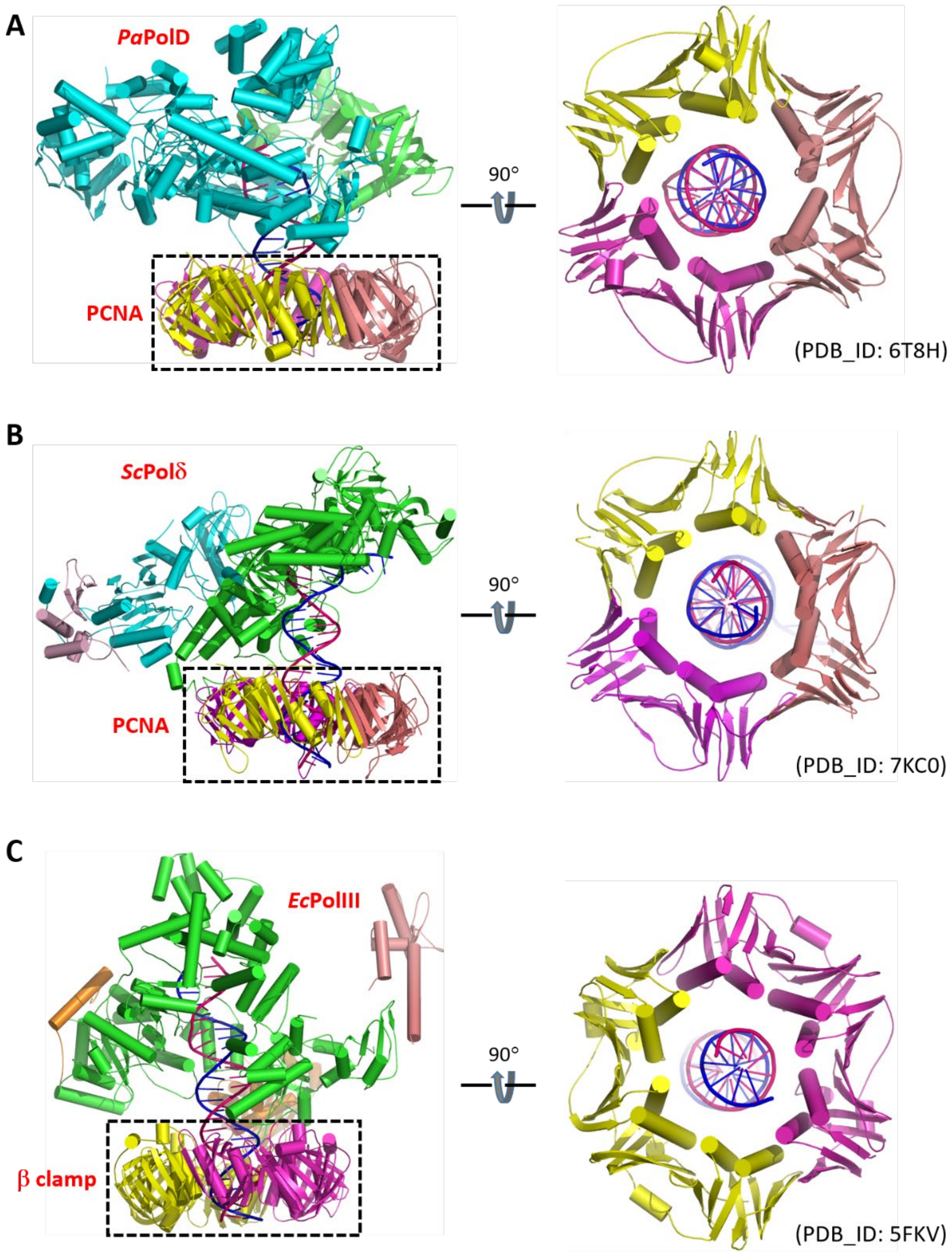

Fig. S10: DNA binding by sliding clamp family proteins in reported structures. (A) Cryo-EM structure of *Pa*PolD in complex with *Pa*PCNA and DNA. (**B**) Cryo-EM structure of *Sc*Polδ in complex with *Sc*PCNA and DNA. (**C**) Cryo-EM structure of *Ec*PolIII-β clamp-DNA ternary complex (PDB_ID: 5FKV). For clarity, polymerase is omitted in the right panel of each structure.

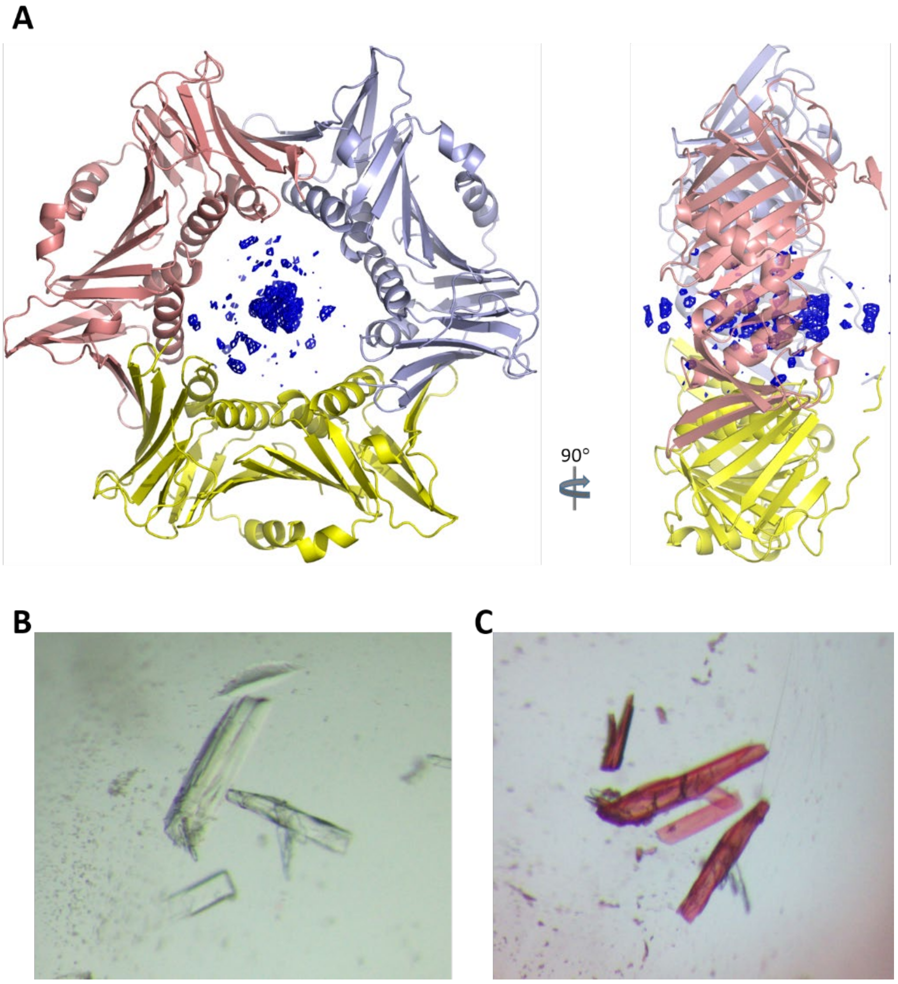

Fig. S11: (**A**) The extra F_o-F_c electron density maps (contour lever, 2.5 σ) observed in Form II *Asfv*PCNA structure. (**B-C**) Image of the *Asfv*PCNA crystals prior to and after staining by GelRed, respectively.

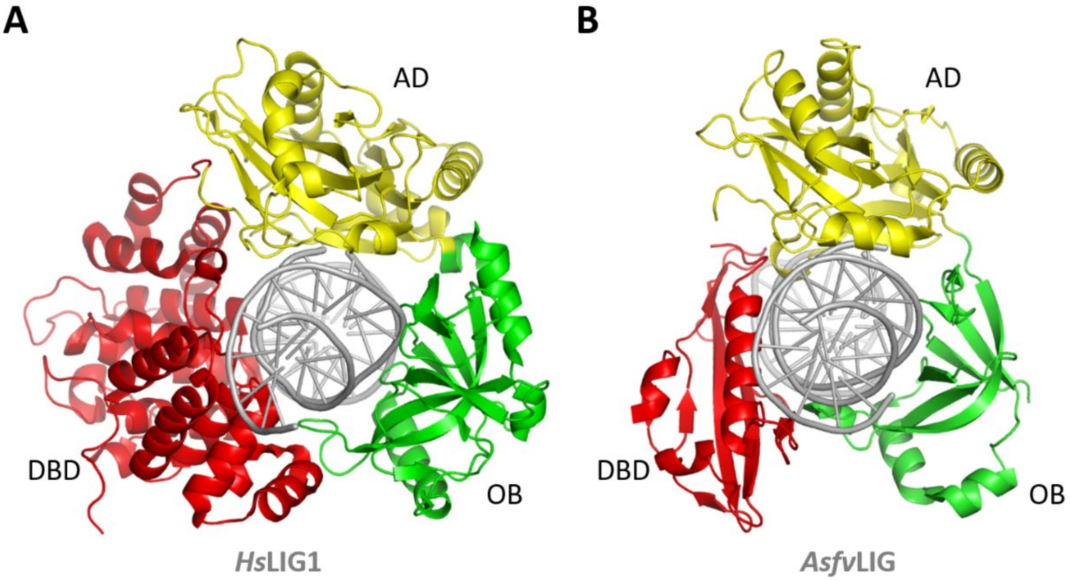

Fig. S12: Structural comparison of (A) *Hs*LIG1 and (B) *Asfv*LIG. The DBD, AD and OB domains are colored in red, yellow and green, respectively. DNA is colored in grey.

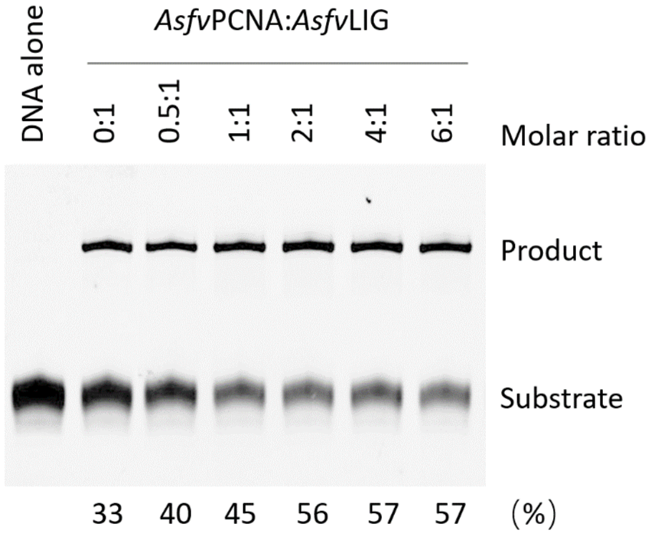

Fig. S13: *In vitro* assays showing the impacts of *Asfv*PCNA on DNA ligation catalyzed by *Asfv*LIG. The percentage of the product is given at the bottom of the image.

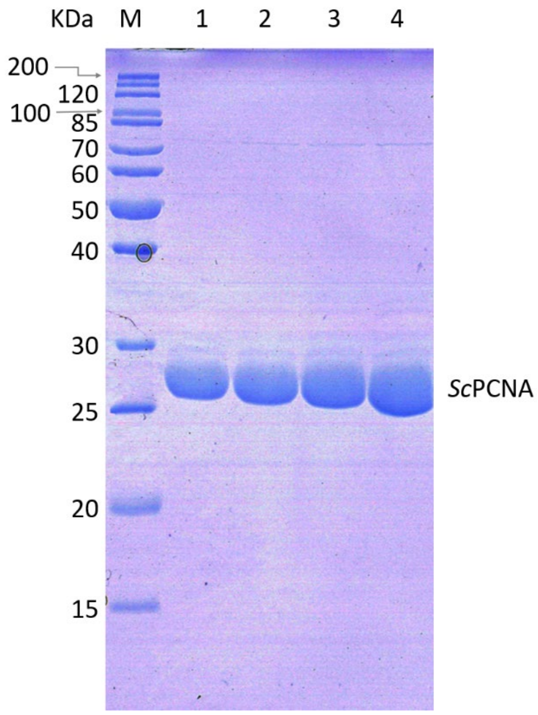

Fig. S14: SDS-PAGE gel analysis of *Sc*PCNA. M, protein marker; lanes 1-4, *Sc*PCNA.

Table S1: Coden-optimized cDNA sequence of *Asfv*PCNA

**The optimized cDNA sequence of *Asfv*PCNA** (from 5' to 3') [a]
GGATCCATGTCTGAGGACATTCGCCGTGGTCCGGGTCGTCCGCCGAAAAAGCGTGTTGTTCCGAACT<br>TCGAACGCAAAGGCATCCTGGAAAAACCGGTCCGTCCGCAGTCTCGTCTGGAATTTTCTTACGACAA<br>CCCGCTCATCTTCAAAAATCTGTTCATCTACTTCAAGAACCTGAAATCTAAAAACATCCTCGTTCGT<br>TGCACCCCGACCGAAATTACCTTCTTCTCTCGTGACCAGTCTCAGGCGTCTTTCGTTATCGCGACCA<br>TCGACGGTAAAAACGTAAACCACTACTACGCGTCTGACGTGTTCTGGCTGGGTATCAACCGTGAACT<br>GGTTGAAAAAATGTTCAATTCTATCGACCGTTCTTTTCTGAAGATTACCATCGTTCACCGCTATGAC<br>AAACCGGAGACCCTCTTCTTCATTTTCACCGACTTCGACATCGACAAAGAATGCACGTACCAGATCA<br>CCGTTTCTGAGCCGGAACTGGACATGGATCTGATTGAAATGGAAAAGTCCATCAGCGAAGAACGCCT<br>CAAAAACTATCCTCTCCGTTGGGAGTTCACCTCTAAACAGCTCAAGAAGACGTTCTCCGACCTGTCT<br>AACTACACCGAACTCGTTACCATTGAGAAACTGGGCGGTGACACTCCGCTGCACCTGTACTTCCAGA<br>AATTCAACTCTATCTCTTACCACGAAATGTACAAATCTTCTAACAAAATCAATCTGACCTCTACCAT<br>CCCGAAATCTCAGGTGTTCCAAATCAACGTTAAAATCGCGCACATCAAATCTCTGGCGTCTGCGATG<br>GTTACTGACAAGATCCGTATCCTGTGTGAAGAAAACGGTAACCTGATCTTTCAGTCTGAGATGGATG<br>CACTCATGCTGAACACCATCACCCTCAACAATACGATCTAACTCGAG

[a]: ***GGATCC*** and ***CTCGAG*** at the 5'-end and 3'-end are BamHI and XhoI recognition sequence.

Table S2: Coden-optimized cDNA sequence of *Sc*PCNA

**The optimized cDNA sequence of *Asfv*PCNA** (from 5' to 3') [a]
GGATCCGGTGGTGGTATGCTGGAAGCAAAATTTGAAGAAGCAAGTCTGTTTAAACGTATTATTGATG<br>GTTTTAAAGATTGTGTGCAGCTGGTTAATTTTCAGTGTAAAGAAGATGGTATCATCGCACAGGCAGT<br>TGATGATAGTCGTGTTCTGCTGGTTAGCCTGGAAATTGGTGTTGAAGCATTTCAGGAATATCGTTGT<br>GATCATCCGGTTACCCTGGGTATGGATCTGACAAGCCTGAGTAAAATTCTGCGTTGTGGTAATAATA<br>CCGATACCCTGACTCTGATTGCGGATAATACCCCGGATAGCATTATTCTGCTGTTTGAAGATACCAA<br>GAAGGATCGTATTGCAGAATATAGCCTGAAACTGATGGATATTGATGCAGATTTTCTGAAAATCGAA<br>GAACTGCAGTATGATAGCACGCTGAGCCTGCCGTCCAGCGAATTTTCAAAAATTGTACGTGATCTGA<br>GTCAGCTGAGCGATTCTATTAATATTATGATTACCAAAGAAACAATCAAATTTGTTGCCGATGGTGA<br>TATTGGTAGCGGTAGCGTTATTATTAAACCGTTTGTGGATATGGAACATCCGGAAACTTCAATTAAA<br>CTGGAAATGGATCAGCCGGTGGATCTGACCTTTGGTGCGAAATATCTGCTGGATATTATTAAAGGTA<br>GCAGCCTGAGCGATCGTGTGGGTATTCGTCTGAGCAGCGAAGCCCCGGCACTGTTTCAGTTTGATCT<br>GAAATCTGGTTTTCTGCAGTTCTTCCTGGCCCCTAAATTTAACGATGAATAACTCGAG

[a]: ***GGATCC*** and ***CTCGAG*** at the 5'-end and 3'-end are BamHI and XhoI recognition sequence.
